# Immunohistochemical Analysis of Toll-Like Receptors, MyD88, and TRIF in Human Papillary Thyroid Carcinoma and Anaplastic Thyroid Carcinoma

**DOI:** 10.1155/2021/4226491

**Published:** 2021-07-01

**Authors:** Yasuhiro Nihon-Yanagi, Megumi Wakayama, Naobumi Tochigi, Fumi Saito, Hideaki Ogata, Kazutoshi Shibuya

**Affiliations:** ^1^Department of Surgical Pathology, Toho University, Omori Hospital, 6-11-1 Omori Nishi, Ota-Ku, Tokyo 143-8541, Japan; ^2^Division of Breast and Endocrine Surgery, Department of Surgery, Toho University, Omori Hospital, 6-11-1 Omori Nishi, Ota-Ku, Tokyo 143-8541, Japan

## Abstract

**Purpose:**

We hypothesized that innate immune response pathways might be involved in thyroid carcinogenesis. To investigate this hypothesis, we aimed at analyzing the expression of several receptors and molecules in the innate immune system in papillary thyroid carcinoma (PTC) and anaplastic thyroid carcinoma (ATC) tissues.

**Methods:**

Of the surgically resected specimens, 11 ATC tissues, 25 PTC tissues, and 8 nodular hyperplasia (NH) tissues were selected and examined for the expression of toll-like receptor (TLR) 2, TLR3, TLR4, TLR5, TLR7, TLR9, the myeloid differentiation primary response gene 88 (MyD88), and toll-interleukin-1 receptor domain-containing adaptor inducing INF-*β* (TRIF) by immunohistochemistry (IHC).

**Results:**

Several TLRs were expressed in each tissue. TLR3 was strongly expressed in all tissues. In contrast, TLR4 was not detected in any tissues. While TLR5 was moderately expressed in NH but significantly reduced in PTC and ATC, TLR9 was absent in NH tissue but moderately expressed in both PTC and ATC. On MyD88 expression, no significant difference was found between PTC and ATC. TRIF was significantly upregulated in PTC and ATC compared to NH. Surprisingly, PTC and ATC tissues exhibited similar expression patterns of TLRs, MyD88, and TRIF.

**Conclusion:**

These data suggest the involvement of the innate immune system in both PTC and ATC. Specifically, TLR3-mediated TRIF activation was confirmed in PTC and ATC. This provides new insight into thyroid carcinogenesis.

## 1. Introduction

Innate immune responses begin with the recognition of microbial components or endogenous molecules released from damaged cells by pattern-recognition receptors, such as toll-like receptors (TLRs) [1–3]. To date, a total of 10 human and 13 murine TLRs have been identified [1–3]. TLRs are expressed on various immune cells, such as dendritic cells (DCs) and macrophages, as well as nonimmune cells, including epithelial cells [1–3]. Signal transduction by TLRs is carried out via two major downstream pathways: the myeloid differentiation primary response gene 88- (MyD88-) dependent and toll-interleukin-1 receptor domain-containing adaptor inducing INF-*β*- (TRIF-) dependent pathways. These pathways ultimately activate transcription factors, such as nuclear factor kappa B (NF-*κ*B) or interferon regulatory factor 3 (IRF3), resulting in the production of various proinflammatory cytokines and type I interferon (IFN) [1–3]. It is currently accepted that chronic inflammation, such as via persistent infection, can promote carcinogenesis. Specifically, persistent infection with *Helicobacter pylori*, hepatitis B or C virus, or human papillomavirus increases the risk of gastric, hepatic, and cervical cancer, respectively [4–6]. Recent research has focused on the relationship between TLRs and different types of carcinoma, such as ovary [7, 8], esophagus [9, 10], lung [11–13], stomach [14], breast [15], colon [16, 17], and many other organs [18–20].

Carcinomas of the thyroid include papillary thyroid carcinoma (PTC), accounting for nearly 90% of all thyroid carcinomas with a favorable prognosis, and anaplastic thyroid carcinoma (ATC), representing only a small percent of all thyroid carcinomas with low survival rate [21, 22]. While the 10-year survival rate of PTC is 95%, a minority of cases have an aggressive course with recurrence and metastases [22, 23]. In addition to infection, the thyroid gland is sensitive to radiation, which may contribute to persistent inflammation of the thyroid. Radiation exposure at ≤18 years of age is a strong risk factor for thyroid cancer, and the risk increases linearly with the dose [24, 25]. Recent studies suggest that TLR agonists and antagonists reduce radiation damage and are more protective with fewer side effects than traditional radioprotective drugs [25–30]. Therefore, TLRs are radioprotective candidates against ionizing radiation.

Based on these findings, we hypothesize that TLRs are related to radiation-induced thyroid carcinogenesis. In this study, we analyzed the expressions of TLR2, TLR3, TLR4, TLR5, TLR7, TLR9, MyD88, and TRIF by immunohistochemistry (IHC) to determine whether there is an association between thyroid carcinoma and innate immune responses. In addition, we compared these innate immune responses in PTC and ATC tissues.

## 2. Materials and Methods

### 2.1. Patients and Tissue Samples

A total of 703 thyroid tissue specimens, including cancerous and nodular hyperplasia (NH), were collected by surgical resection at the Department of Surgery, Toho University Omori Medical Center (Tokyo, Japan), between 2001 and 2019. Of the specimens, 11 ATC tissues were diagnosed histologically. To avoid any bias, 25 PTC tissues were randomly selected, and 8 NH tissues were used as a noncancerous control. Of the PTC samples, one case coexisted with Hashimoto's thyroiditis. PTC samples were classified using the TMN staging system [31] as follows: stage I (*n* = 19), stage II (*n* = 3), stage III (*n* = 2), and stage IVb (*n* = 1). Patient characteristics are summarized in Table 1.

### 2.2. Immunohistochemistry

A polymer-based detection technique was used to prevent endogenous avidin and biotin activity in the thyroid gland epithelium [32]. All samples were formalin-fixed and paraffin-embedded. Tissue sections were deparaffinized in xylene and then rehydrated with a gradient ethanol series. For antigen retrieval, sections were treated in Target Retrieval Solution (TRS), pH 9 (Nichirei Biosciences, Inc., Tokyo, Japan), in a water bath at 98°C for 40 min. Slides were then cooled at room temperature and washed in phosphate-buffered saline (PBS). Next, the sections were treated with 0.3% H_2_O_2_ in methanol for 10 min, washed in PBS, and then incubated with primary antibodies for 60 min at room temperature. Sections were then washed in PBS twice and incubated with horseradish peroxidase-labeled polymer solution (Nichirei Biosciences, Inc., Tokyo, Japan) for 30 min at room temperature. Antigen visualization was performed using 3,3-diaminobenzidine tetrahydrochloride (DAB; Dojindo Laboratories, Kumamoto, Japan). Finally, sections were counterstained with hematoxylin, dehydrated with ethanol, and mounted.

### 2.3. Antibodies

Antibodies specific for TLR2 (ab213676), TLR3 (ab62566), and TRIF (ab13810) were from Abcam. Antibodies specific for TLR4 (HPA049174), TLR5 (HPA015573), TLR7 (HPA059613), and TLR9 (HPA004731) were from Sigma-Aldrich. Antibodies specific for MyD88 (sc-136970) were from Santa Cruz Biotechnology.

### 2.4. Data Scoring Methods

Immunohistochemical expression of TLRs was scored by two independent pathologists following a previously described scoring system [10, 11, 14, 15, 33, 34]. Staining intensity was scored as follows: 0, no staining; 1, weak staining; 2, moderate staining; and 3, intense staining. If there was a discrepancy between the scores of both pathologists, a third pathologist scored the tissues to gain consensus. The staining proportion was scored as the percentage of the positive cell area (0–100%). The overall IHC score was calculated by multiplying the intensity score by the percentage of positive staining, resulting in values ranging from 0 to 300.

### 2.5. Statistical Analysis

Statistical analyses were performed using a bell curve in Excel (Social Survey Research Information Co., Ltd. Tokyo, Japan). IHC scores were compared between groups using Kruskal-Wallis and Steel-Dwass tests. *p* values <0.05 were considered statistically significant.

## 3. Results

### 3.1. TLR Expression in PTC, ATC, and NH Tissues

**Figure 1:**
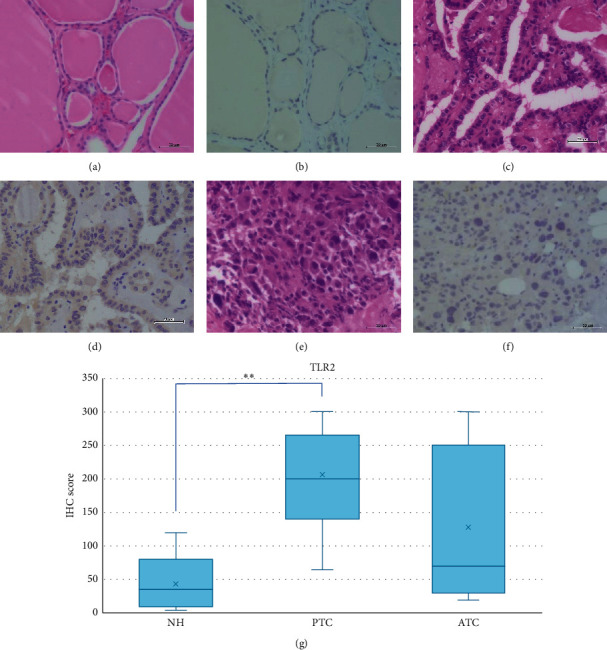
Representative images of TLR2 expression in NH, PTC, and ATC. HE: hematoxylin and eosin staining; IHC: immunohistochemistry; NH: nodular hyperplasia; PTC: papillary thyroid carcinoma; ATC: anaplastic thyroid carcinoma. Scale bar: 50 *μ*m. (a) HE of NH. (b) Weak intensity of IHC for TLR2 in NH. (c) HE of PTC. (d) Moderate intensity of IHC for TLR2 in PTC. (e) HE of ATC. (f) Moderate intensity of IHC for TLR2 in ATC. (g) TLR2 IHC scores in NH, PTC, and ATC. The IHC score was only significantly higher in PTC compared to NH (*p* < 0.001). ^*∗∗*^*p* < 0.001.

We observed weak TLR2 staining in NH but moderate staining in PTC and ATC tissues (Figures 1(a)–1(f)).

However, the IHC score was only significantly higher in PTC compared to NH (*p* < 0.001) (Figure 1(g)).

**Figure 2:**
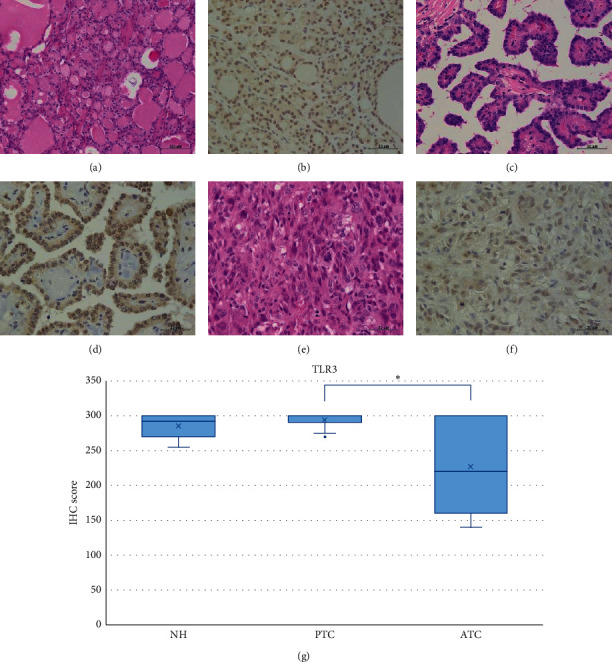
Representative images of TLR3 expression in NH, PTC, and ATC. All tissues exhibited strong TLR3 staining within the cytoplasm or partially in the nucleus. HE: hematoxylin and eosin staining; IHC: immunohistochemistry; NH: nodular hyperplasia; PTC: papillary thyroid carcinoma; ATC: anaplastic thyroid carcinoma. Scale bar: 50 *μ*m. (a) HE of NH. (b) High intensity of IHC for TLR3 in NH. (c) HE of PTC. (d) High intensity of IHC for TLR3 in PTC. (e) HE of ATC. (f) High intensity of IHC for TLR3 in ATC. (g) TLR3 IHC scores in NH, PTC, and ATC. The IHC expression score was significantly higher in PTC compared to ATC (*p* < 0.05). ^*∗*^*p* < 0.05.

**Figure 3:**
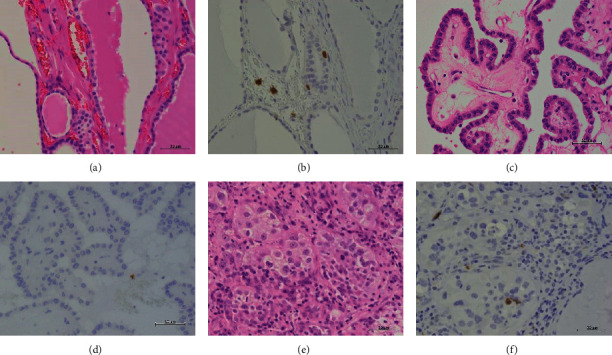
Representative images of TLR4 expression in NH, PTC, and ATC. No expression was detected, except for mononuclear cells present in the stroma. HE: hematoxylin and eosin staining; IHC: immunohistochemistry; NH: nodular hyperplasia; PTC: papillary thyroid carcinoma; ATC: anaplastic thyroid carcinoma. Scale bar: 50 *μ*m. (a) HE of NH. (b) No expression of IHC for TLR4 in NH. (c) HE of PTC. (d) No expression of IHC for TLR4 in PTC. (e) HE of ATC. (f) No expression of IHC for TLR4 in ATC.

**Figure 4:**
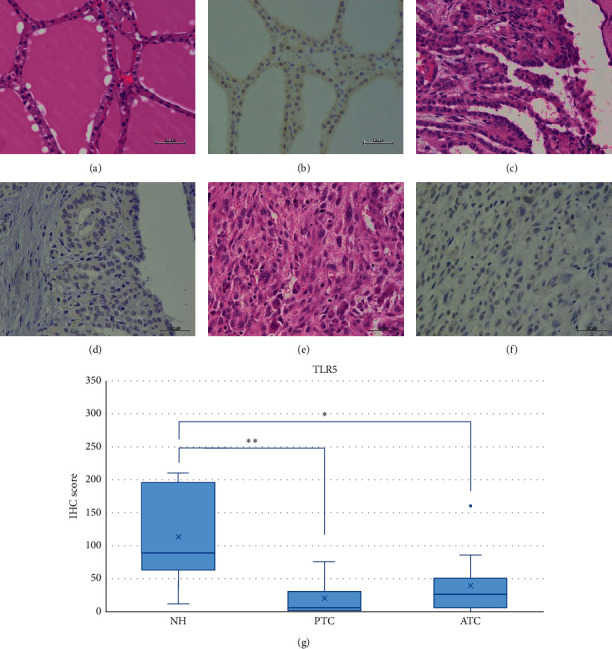
Representative images of TLR5 expression in NH, PTC, and ATC. Moderate TLR5 staining was identified in NH in the cytoplasm or partially in the nucleus. Reduced expression was observed in PTC and ATC. HE: hematoxylin and eosin staining; IHC: immunohistochemistry; NH: nodular hyperplasia; PTC: papillary thyroid carcinoma; ATC: anaplastic thyroid carcinoma. Scale bar: 50 *μ*m. (a) HE of NH. (b) Moderate intensity of IHC for TLR5 in NH. (c) HE of PTC. (d) Weak intensity of IHC for TLR5 in PTC. (e) HE of ATC. (f) Weak intensity of IHC for TLR5 in ATC. (g) TLR5 IHC scores in NH, PTC, and ATC. The IHC scores were significantly reduced in PTC and ATC compared to NH (*p* < 0.001 and *p* < 0.05, respectively). ^*∗∗*^*p* < 0.001 and ^*∗*^*p* < 0.05.

**Figure 5:**
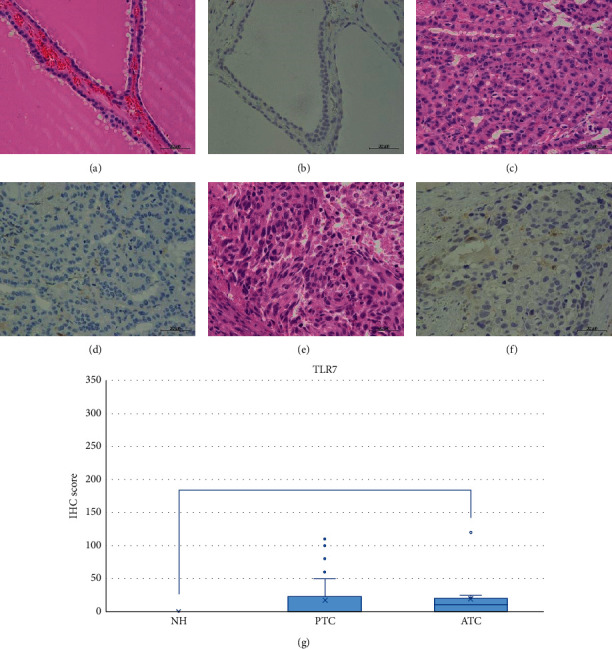
Representative images of TLR7 expression in NH, PTC, and ATC. TLR7 expression was virtually undetectable in NH and weak in PTC and ATC. HE: hematoxylin and eosin staining; IHC: immunohistochemistry; NH: nodular hyperplasia; PTC: papillary thyroid carcinoma; ATC: anaplastic thyroid carcinoma. Scale bar: 50 *μ*m. (a) HE of NH. (b) No expression of IHC for TLR7 in NH. (c) HE of PTC. (d) Quit weak intensity of IHC for TLR7 in PTC. (e) HE of ATC. (f) Quit weak intensity of IHC for TLR7 in ATC. (g) TLR7 IHC scores in NH, PTC, and ATC. The IHC score was significantly upregulated in ATC compared to NH (*p* < 0.001). ^*∗∗*^*p* < 0.001.

**Figure 6:**
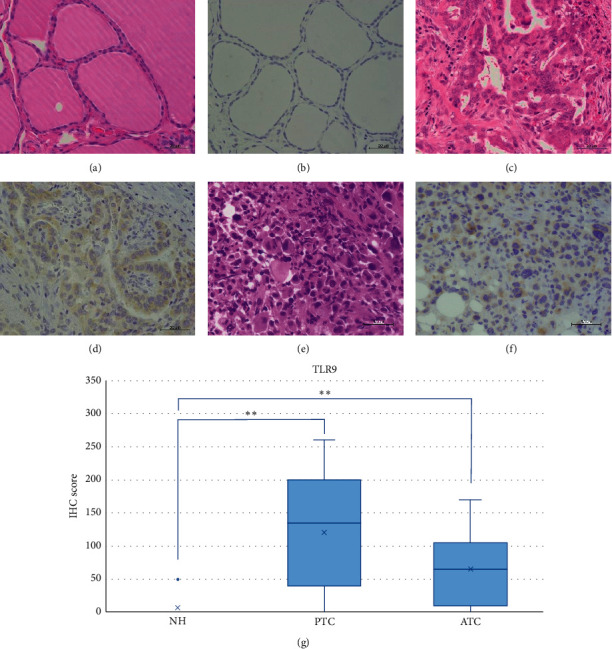
Representative images of TLR9 expression in NH, PTC, and ATC. TLR9 staining was absent in NH. Moderate cytoplasmic expression was observed in PTC and ATC. HE: hematoxylin and eosin staining; IHC: immunohistochemistry; NH: nodular hyperplasia; PTC: papillary thyroid carcinoma; ATC: anaplastic thyroid carcinoma. Scale bar: 50 *μ*m. (a) HE of NH. (b) No expression of IHC for TLR9 in NH. (c) HE of PTC. (d) Moderate intensity of IHC for TLR9 in PTC. (e) HE of ATC. (f) Moderate intensity of IHC for TLR9 in ATC. (g) TLR9 IHC scores in NH, PTC, and ATC. The IHC scores were significantly upregulated in PTC and ATC compared to NH (*p* < 0.001). ^*∗∗*^*p* < 0.001.

NH, PTC, and ATC tissues exhibited strong TLR3 staining in the cytoplasm or partially in the nucleus (Figures 2(a)–2(f)). The IHC expression score was significantly higher in PTC compared to ATC (*p* < 0.05) (Figure 2(g)). In contrast, we did not observe TLR4 expression in the NH, PTC, or ATC tissues, except for mononuclear cells present in the stroma (Figures 3(a)–3(f)). Moderate TLR5 expression was found in NH tissues in the cytoplasm or partially in the nucleus (Figures 4(a)–4(f)). TLR5 expression and the IHC score were significantly reduced in PTC and ATC compared to NH (*p* < 0.001 and *p* < 0.05, respectively) (Figure 4(g)). TLR7 expression was virtually undetectable in NH and relatively weak in PTC and ATC (Figures 5(a)–5(f)). However, the IHC score was significantly upregulated in ATC compared to NH (*p* < 0.001) (Figure 5(g)). Moderate cytoplasmic TLR9 staining was observed in PTC and ATC but not NH (Figures 6(a)–6(f)). The IHC score was significantly upregulated in PTC and ATC compared to NH (*p* < 0.001 and *p* < 0.05, respectively) (Figure 6(g)).

### 3.2. Expression of Downstream TLR-Signaling Molecules in PTC, ATC, and NH Tissues

**Figure 7:**
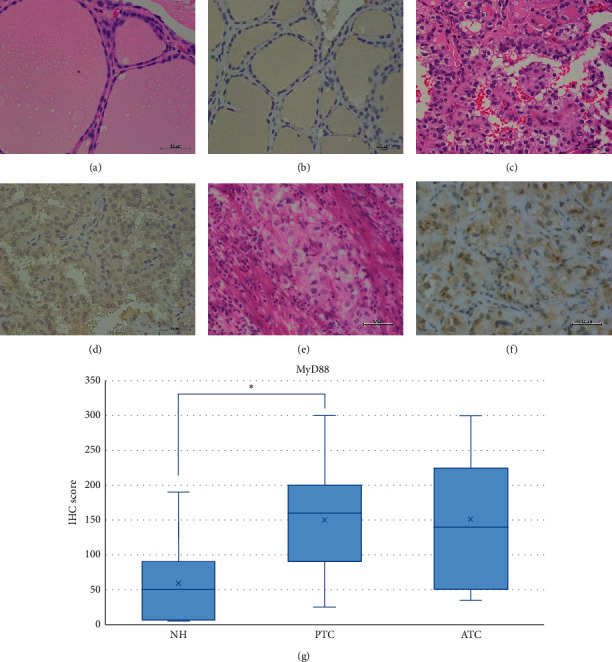
Representative images of MyD88 expression in NH, PTC, and ATC. Brown pigmentation was prominent in the colloid. Weak MyD88 expression was observed in NH, and moderate expression was detected in PTC and ATC. HE: hematoxylin and eosin staining; IHC: immunohistochemistry; NH: nodular hyperplasia; PTC: papillary thyroid carcinoma; ATC: anaplastic thyroid carcinoma. Scale bar: 50 *μ*m. (a) HE of NH. (b) Weak intensity of IHC for MyD88 in NH. (c) HE of PTC. (d) Moderate intensity of IHC for MyD88 in PTC. (e) HE of ATC. (f) Moderate intensity of IHC for MyD88 in ATC. (g) MyD88 IHC scores in NH, PTC, and ATC. The IHC score was significantly higher in PTC compared to NH (*p* < 0.05). ^*∗*^*p* < 0.05.

**Figure 8:**
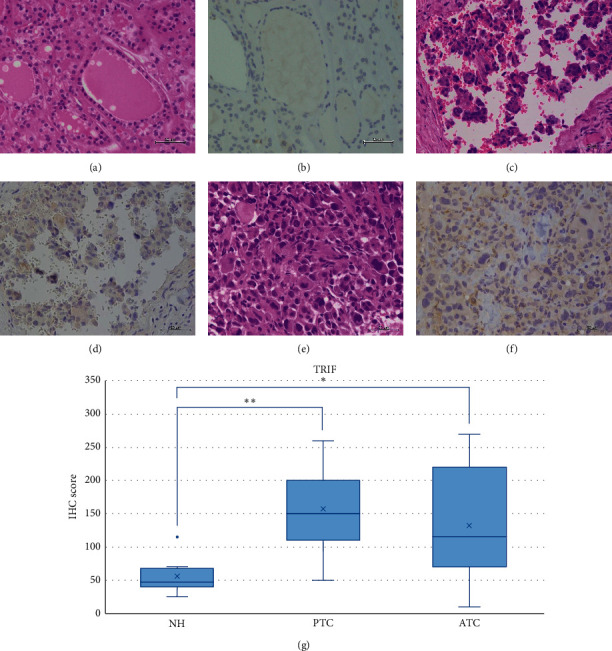
Representative images of TRIF expression in NH, PTC, and ATC. Weak TRIF expression was observed in NH, and moderate expression was detected in PTC and ATC. HE: hematoxylin and eosin staining; IHC: immunohistochemistry; NH: nodular hyperplasia; PTC: papillary thyroid carcinoma; ATC: anaplastic thyroid carcinoma. Scale bar: 50 *μ*m. (a) HE of NH. (b) Weak intensity of IHC for TRIF in NH. (c) HE of PTC. (d) Moderate intensity of IHC for TRIF in PTC. (e) HE of ATC. (f) Moderate intensity of IHC for TRIF in ATC. (g) TRIF IHC score values in NH, PTC, and ATC. The IHC scores were significantly higher in PTC and ATC compared to NH, respectively (*p* < 0.001 and *p* < 0.05, respectively). ^*∗∗*^*p* < 0.001 and ^*∗*^*p* < 0.05.

We observed prominent brown pigmentation in the colloid that mimics the strong MyD88 staining on thyroid gland epithelium. However, only weak positivity of MyD88 was observed in NH and moderate staining in PTC and ATC (Figures 7(a)–7(f)). The IHC score was significantly higher in PTC compared to NH (*p* < 0.05) (Figure 7(g)). Similarly, we observed weak positivity of TRIF in NH and moderate staining in PTC and ATC (Figures 8(a)–8(f)), with a significantly higher IHC score in PTC and ATC compared to NH (*p* < 0.001 and *p* < 0.05, respectively) (Figure 8(g)).

## 4. Discussion

In this study, we found differential expression of TLRs in NH, PTC, and ATC tissues. Furthermore, TRIF was significantly upregulated in both carcinoma tissues compared to the noncarcinoma control. These results suggest that innate immune responses may be involved in thyroid carcinogenesis.

TLR9 expression was significantly increased in both PTC and ATC but absent in NH. TLR2 and TLR7 were also observed in PTC and ATC, although TLR7 expression was low. In contrast, we observed moderate expression of TLR5 in NH and downregulation in both PTC and ATC.

MyD88 was also significantly upregulated in PTC compared to NH. However, no significant differences were found between NH and ATC and between PTC and ATC. All TLRs, except for TLR3, initiate MyD88-dependent signaling. Although we observed almost identical TLR expression patterns in these carcinoma tissues, the contribution of TLRs to the MyD88-dependent pathway in PTC and ATC remains unclear, and more complicated mechanisms may occur following TLR signaling and activation of the MyD88-dependent pathway.

Interestingly, we observed a significant upregulation of TRIF in PTC and ATC compared with NH. TLR3 was strongly expressed in NH, PTC, and ATC tissues. In contrast, no cancer tissues expressed TLR4. Consistent with our findings, previous reports demonstrate overexpression of TLR3 in human normal thyroid gland epithelium, PTC tissues, and the PTC cell line [21, 35]. As the TRIF-dependent pathway is activated via TLR3 or TLR4, upregulation of TRIF in PTC and ATC results in TLR3 activation.

Takemura et al. [28] demonstrated that total body irradiation of mice specifically activated TLR3 on crypt epithelial cells in the small intestine, which exacerbated severe intestinal injury through the TRIF pathway. Moreover, TlR3-/- mice were resistant to this type of injury. Radiation is a strong risk factor for thyroid cancer [24, 25], which may directly or indirectly stimulate TLRs by releasing molecules from injured cells. Therefore, radiation may cause chronic damage in the thyroid tissue via activation of TLRs, contributing to carcinogenesis. We hypothesize that TLR3 may be a candidate sensor for radiosensitivity, resulting in activation of the TRIF-dependent pathway; however, this hypothesis needs to be fully explored.

Harii et al. [35] demonstrated TLR3 overexpression in human thyrocytes isolated from patients with the autoimmune thyroid disease Hashimoto's thyroiditis. Although several studies have investigated the link between Hashimoto's thyroiditis and thyroid carcinoma, the results are still controversial [36, 37]. In our sample, we found only one case of Hashimoto's thyroiditis in a patient with PTC as described in “Materials and Methods.” Therefore, we could not determine the relationship between autoimmunity and thyroid carcinoma in the present study.

In addition to viral double-stranded RNA (dsRNA), TLR3 can recognize self-nucleic acids released upon cell death [38]. Activation of the TRIF-dependent pathway results in the activation of IFN-regulatory factor-3 (IRF-3) and release of several inflammatory cytokines and type I IFN [1, 2, 39]. Therefore, blocking DAMPs (e.g., derived from radiation-injured cells) detected by TLR3 may be a more specific treatment than using conventional TLR3 antagonists and may inhibit TRIF or IRF-3 activation or release of TRIF-dependent cytokines and IFNs.

Some cases of PTC have recurrence and may become life-threatening. However, the vast majority of PTC cases in this study were in the early stage (76% were stage I using TMN classification) because of a paucity of aggressive cases. Therefore, we predicted that PTC and ATC tissues would exhibit different TLR expression patterns due to the distinct clinicopathological features. However, TLR, TRIF, and MyD88 expressions were similar in both cancer tissues. McCall et al. [21] Previously compared the expression of TLR3 and noncanonical Wnt5a between PTC and ATC cell lines and found that the noncanonical, and not canonical, Wnt pathway contributes to PTC. Based on this study, other pathways, such as the Wnt pathway, may contribute to the transformation of PTC into ATC. Further studies are needed to investigate this distinct mechanism.

## 5. Conclusion

Some TLRs were expressed in PTC and ATC. Furthermore, TRIF, one of the essential molecules in the two major downstream TLR signaling pathways, was upregulated in both PTC and ATC compared to NH. Together, our data suggest that innate immune responses may contribute to thyroid tumorigenesis. Specifically, our data suggest activation of the TRIF-dependent pathway by TLR3 in thyroid carcinoma, which may be an essential receptor for radiation damage.

## Figures and Tables

**Table 1:** Characteristics of patients.

PTC (*n* = 25)	
Age	24–76 years (median: 48 years)
Sex	Female: 15; male: 10
Tumor size (mm)	5–150 (average: 27.1)
Number of positive nodes	0–24 (average: 4.24)
Stage (UICC/TMN)	Stage I (19); stage II (3); stage III (2); stage IV (1)

ATC (*n* = 11)	
Age	51–85 years (median: 73 years)
Sex	Female: 5; male: 6

NH (*n* = 8)	
Age	22–68 years (median: 60 years)
Sex	Female: 3; male: 5

## Data Availability

All data generated or analysed during this study are included in the article and in the Supplementary Materials.

## Supplementary Materials

Supplementary Materials Figure S-1-1: HE and IHC images of NH, sample 1. Figure S-1-2: HE and IHC images of NH, sample 2. Figure S-1-3: HE and IHC images of NH, sample 3. Figure S-1-4: HE and IHC images of NH, sample 4. Figure S-1-5: HE and IHC images of NH, sample 5. Figure S-1-6: HE and IHC images of NH, sample 6. Figure S-1-7: HE and IHC images of NH, sample 7. Figure S-1-8: HE and IHC images of NH, sample 8. Figure S-2-1: HE and IHC images of PTC, sample 1. Figure S-2-2: HE and IHC images of PTC, sample 2. Figure S-2-3: HE and IHC images of PTC, sample 3. Figure S-2-4: HE and IHC images of PTC, sample 4. Figure S-2-5: HE and IHC images of PTC, sample 5. Figure S-2-6: HE and IHC images of PTC, sample 6. Figure S-2-7: HE and IHC images of PTC, sample 7. Figure S-2-8: HE and IHC images of PTC, sample 8. Figure S-2-9: HE and IHC images of PTC, sample 9. Figure S-2-10: HE and IHC images of PTC, sample 10. Figure S-2-11: HE and IHC images of PTC, sample 11. Figure S-2-12: HE and IHC images of PTC, sample 12. Figure S-2-13: HE and IHC images of PTC, sample 13. Figure S-2-14: HE and IHC images of PTC, sample 14. Figure S-2-15: HE and IHC images of PTC, sample 15. Figure S-2-16: HE and IHC images of PTC, sample 16. Figure S-2-17: HE and IHC images of PTC, sample 17. Figure S-2-18: HE and IHC images of PTC, sample 18. Figure S-2-19: HE and IHC images of PTC, sample 19. Figure S-2-20: HE and IHC images of PTC, sample 20. Figure S-2-21: HE and IHC images of PTC, sample 21. Figure S-2-22: HE and IHC images of PTC, sample 22. Figure S-2-23: HE and IHC images of PTC, sample 23. Figure S-2-24: HE and IHC images of PTC, sample 24. Figure S-2-25: HE and IHC images of PTC, sample 25. Figure S-3-1: HE and IHC images of ATC, sample 1. Figure S-3-2: HE and IHC images of ATC, sample 2. Figure S-3-3: HE and IHC images of ATC, sample 3. Figure S-3-4: HE and IHC images of ATC, sample 4. Figure S-3-5: HE and IHC images of ATC, sample 5. Figure S-3-6: HE and IHC images of ATC, sample 6. Figure S-3-7: HE and IHC images of ATC, sample 7. Figure S-3-8: HE and IHC images of ATC, sample 8. Figure S-3-9: HE and IHC images of ATC, sample 9. Figure S-3-10: HE and IHC images of ATC, sample 10. Figure S-3-11: HE and IHC images of ATC, sample 11.

## References

[1] Akira S., Uematsu S., Takeuchi O. (2006). Pathogen recognition and innate immunity. *Cell*.

[2] Kawai T., Akira S. (2011). Toll-like receptors and their crosstalk with other innate receptors in infection and immunity. *Immunity*.

[3] Rakoff-Nahoum S., Medzhitov R. (2009). Toll-like receptors and cancer. *Nature Reviews Cancer*.

[4] O’Neill L. (2008). Toll-like receptors in cancer. *Oncogene*.

[5] Grivennikov S. I., Greten F. R., Karin M. (2010). Immunity, inflammation, and cancer. *Cell*.

[6] Karin M. (2006). Nuclear factor-kappaB in cancer development and progression. *Nature*.

[7] Zhou M., McFarland-Mancini M. M., Funk H. M. (2009). Toll-like receptor expression in normal ovary and ovarian tumors. *Cancer Immunol Immunother*.

[8] Wang A. C., Ma Y. B., Wu F. X., Ma Z. F., Liu N. F., Gao R. (2014). TLR4 induces tumor growth and inhibits paclitaxel activity in MyD88-positive human ovarian carcinoma in vitro. *Oncology Letters*.

[9] Sheyhidin I., Nabi G., Hasim A. (2011). Overexpression of TLR3, TLR4, TLR7 and TLR9 in esophageal squamous cell carcinoma. *World Journal of gastroenterology*.

[10] Helminen O., Huhta H., Takala H., Lehenkari P. P., Saarnio J., Kauppila J. H. (2014). Increased Tolllike receptor 5 expression indicates esophageal columnar dysplasia. *Virchows Archiv: An International Journal of Pathology*.

[11] Zhou H., Chen J.-h., Hu J. (2014). High expression of Toll-like receptor 5 correlates with better prognosis in non-small-cell lung cancer: an anti-tumor effect of TLR5 signaling in non-small cell lung cancer. *Journal of Cancer Research and Clinical Oncology*.

[12] Chatterjee S., Crozet L., Damotte D. (2014). TLR7 promotes tumor progression, chemotherapy resistance, and poor clinical outcomes in non–small cell lung cancer. *Cancer Research*.

[13] Lan F., Yue X., Ren G., Wang Y., Xia T. (2014). Serum toll-like receptors are potential biomarkers of radiation pneumonia in locally advanced NSCLC. *International Journal of Clinical and Experimental Pathology*.

[14] Fernandez-Garcia B., Eiró N., González-Reyes S. (2014). Clinical significance of Toll-like receptor 3,4, and 9 in gastric cancer. *Journal of Immunotherapy*.

[15] González-Reyes S., Marín L., González L. (2010). Study of TLR3, TLR4 and TLR9 in breast carcinomas and their association with metastasis. *BMC Cancer*.

[16] Nihon-Yanagi Y., Terai K., Murano M., Matsumoto T., Okazumi S. (2012). Tissue expression of Toll-like receptors 2 and 4 in sporadic human colorectal cancer. *Cancer Immunology, Immunotherapy*.

[17] Xiang L., Wang S., Jin X., Duan W., Ding X., Zheng C. (2012). Expression of BMP2, TLR3, TLR4 and COX2 in colorectal polyps, adenoma and adenocarcinoma. *Molecular Medicine Reports*.

[18] Werner J., Decarlo C. A., Escott N., Zehbe I., Ulanova M. (2012). Expression of integrins and Toll-like receptors in cervical cancer: effect of infectious agents. *Innate Immunity*.

[19] Ochi A., Graffeo C. S., Zambirinis C. P. (2012). Toll-like receptor 7 regulates pancreatic carcinogenesis in mice and humans. *J Clin Invest*.

[20] Sun Z., Luo Q., Ye D., Chen W., Chen F. (2012). Role of toll-like receptor 4 on the immune escape of human oral squamous cell carcinoma and resistance of cisplatin-induced apoptosis. *Molecular Cancer*.

[21] McCall K. D., Harii N., Lewis C. J., Malgor R., Kim W. B., Saji M. (2007). High basal levels of functional toll-like receptor 3 (TLR3) and noncanonical Wnt5a are expressed in papillary thyroid cancer and are coordinately decreased by phenylmethimazole together with cell proliferation and migration. *Endocrinology*.

[22] Baloch Z. W., LiVolsi V. A. (2018). Special types of thyroid carcinoma. *Histopathology*.

[23] Ito Y., Kudo T., Takamura Y., Kobayashi K., Miya A., Miyauchi A. (2011). Prognostic significance of carcinoma extension from primary lesions and metastatic nodes in papillary thyroid carcinoma: appropriateness of three subdivisions of extension. *Endocrine Journal*.

[24] Thompson D. E., Mabuchi K., Ron E. (1994). Cancer incidence in atomic bomb survivors. Part II: solid tumors, 1958-1987. *Radiation Research*.

[25] Imaizumi M., Usa T., Tominaga T. (2006). Radiation dose-response relationships for thyroid nodules and autoimmune thyroid diseases in Hiroshima and Nagasaki atomic bomb survivors 55-58 years after radiation exposure. *JAMA*.

[26] Bai H., Sun F., Yang G. (2019). CBLB502, a Toll-like receptor 5 agonist, offers protection against radiation-induced male reproductive system damage in mice. *Biology of Reproduction*.

[27] Liu Z., Lei X., Li X., Cai J. M., Gao F., Yang Y. Y. (2018). Toll-like receptors and radiation protection. *European Review for Medical and Pharmacological Sciences*.

[28] Takemura N., Kawasaki T., Kunisawa J. (2014). Blockade of TLR3 protects mice from lethal radiation-induced gastrointestinal syndrome. *Nature Communications*.

[29] Ratikan J. A., Micewicz E. D., Xie M. W., Schaue D. (2015). Radiation takes its toll. *Cancer Letters*.

[30] Saha S., Bhanja P., Liu L. (2012). TLR9 agonist protects mice from radiation-induced gastrointestinal syndrome. *PLoS One*.

[31] Lloyd R. V., Osamura R. Y., Klöppel G., Rosai J. (2017). *WHO Classification of Tumours of Endocrine Organs*.

[32] Nikiel B., Chekan M., Jarzab M., Lange D. (2009). Endogenous avidin biotin activity (EABA) in thyroid pathology: immunohistochemical study. *Thyroid Research*.

[33] Fedchenko N., Reifenrath J. (2014). Different approaches for interpretation and reporting of immunohistochemistry analysis results in the bone tissue. *Diagnostic Pathology*.

[34] Choudhury K. R., Yagle K. J., Swanson P. E., Krohn K. A., Rajendran J. G. (2010). A robust automated measure of average antibody staining in immunohistochemistry images. *The Journal of Histochemistry and Cytochemistry*.

[35] Harii N., Lewis C. J., Vasko V. (2005). Thyrocytes express a functional toll-like receptor 3: overexpression can Be induced by viral infection and reversed by phenylmethimazole and is associated with hashimoto’s autoimmune thyroiditis. *Molecular Endocrinology*.

[36] Paparodis R., Imam S., Todorova-Koteva K., Staii A., Jaume J. C. (2014). Hashimoto’s thyroiditis pathology and risk for thyroid cancer. *Thyroid*.

[37] Penta L., Cofini M., Lanciotti L., Leonardi A., Principi N., Esposito S. (2018). Hashimoto’s disease and thyroid cancer in children: are they associated?. *Frontiers in Endocrinology*.

[38] Piccinini A. M., Midwood K. S. (2010). DAMPening inflammation by modulating TLR signalling. *Mediators of Inflammation*.

[39] Steinhagen F., Kinjo T., Bode C., Klinman D. M. (2011). TLR-based immune adjuvants. *Vaccine*.

