# Supplementary material for: Immunohistochemical Analysis of Toll-Like Receptors, MyD88, and TRIF in Human Papillary Thyroid Carcinoma and Anaplastic Thyroid Carcinoma
Source: J Thyroid Res. 2021 Jul 1;2021:4226491. doi: 10.1155/2021/4226491 (PMC8270699; doi:10.1155/2021/4226491)

Figure S-1-1: HE and IHC images of NH, sample 1

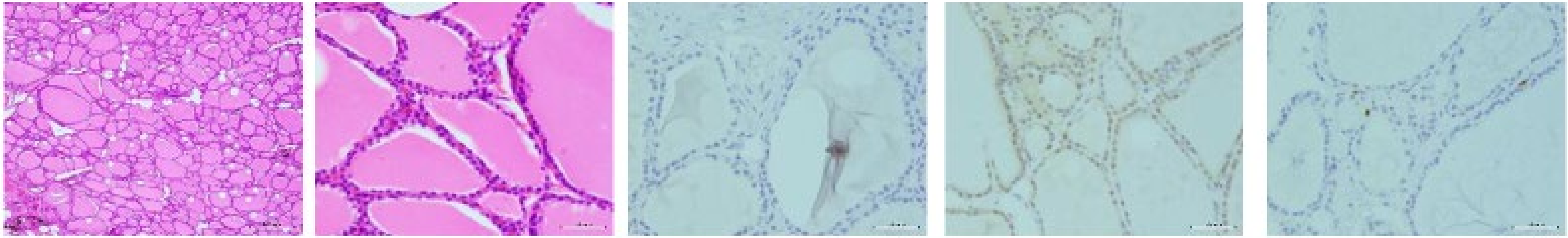

HE, low magnification

HE, high magnification

TLR2 score: 1x20=20

TLR3 score: 3x90=270

TLR4 score: 0

TLR5 score: 1x10+2x50+3x30=200

TLR7 score: 0

TLR9 score: 0

MyD88 score: 1x40+2x30+3x30=190

TRIF score: 1x25=25

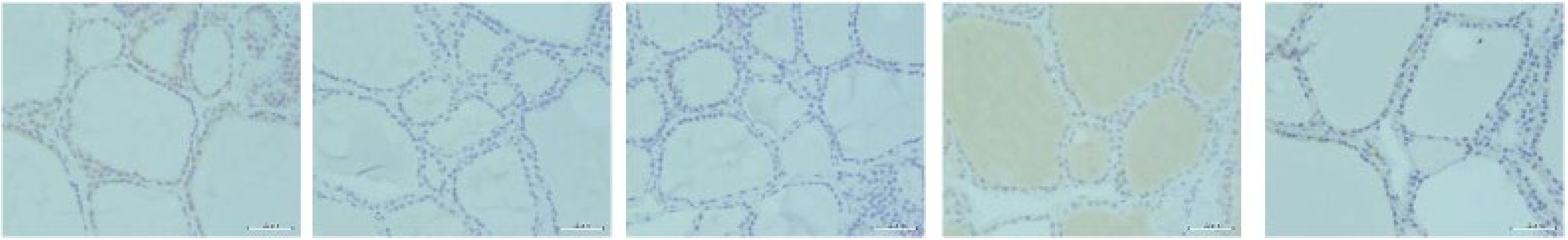

Figure S-1-2: HE and IHC images of NH, sample 2

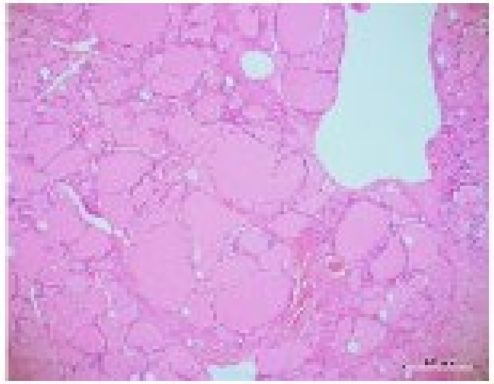
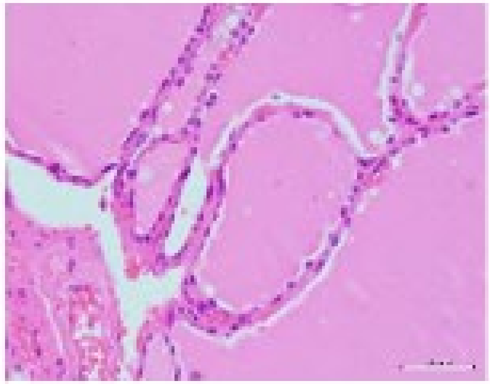
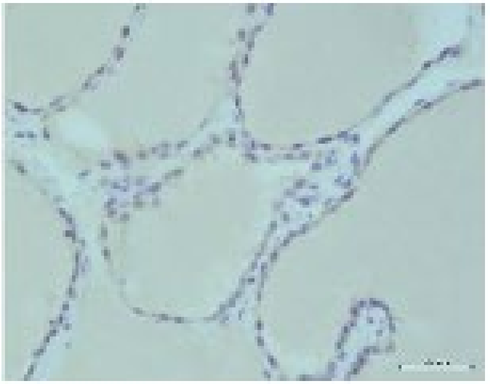
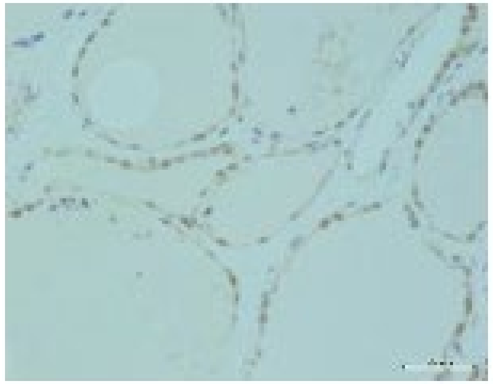
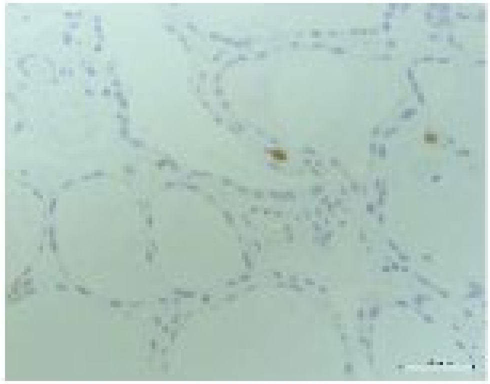

HE, low magnification

HE, high magnification

TLR2 score: 1x30+2x30+3 x10=120

TLR3 score: 3x95=285

TLR4 score: 0

TLR5 score: 1x35+2x20=75

TLR7 score: 0

TLR9 score: 0

MyD88 score: 3x5=15

TRIF score: 3x15=45

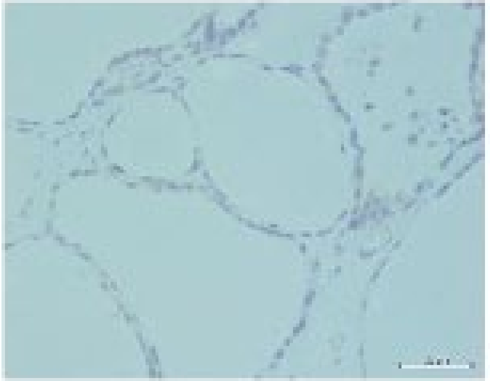
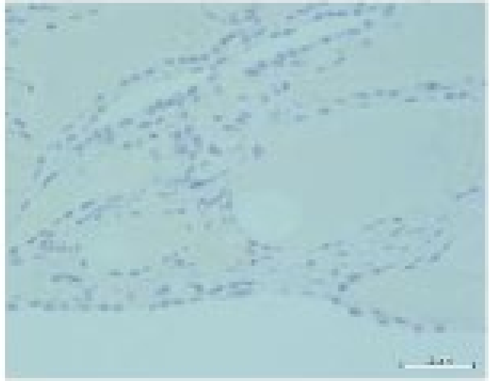
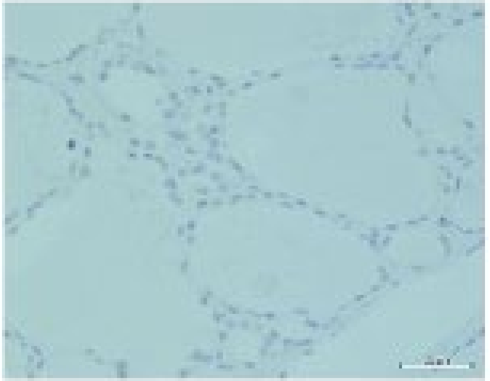
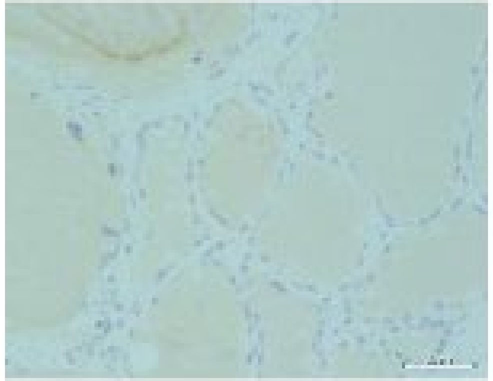
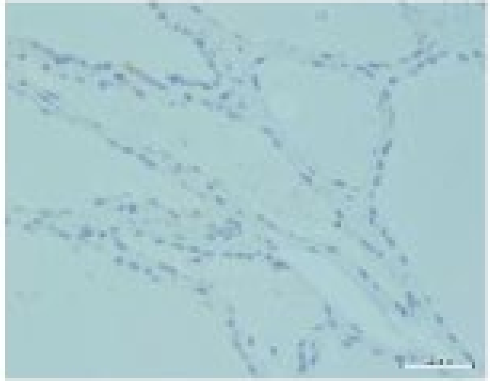

Figure S-1-3: HE and IHC images of NH, sample 3

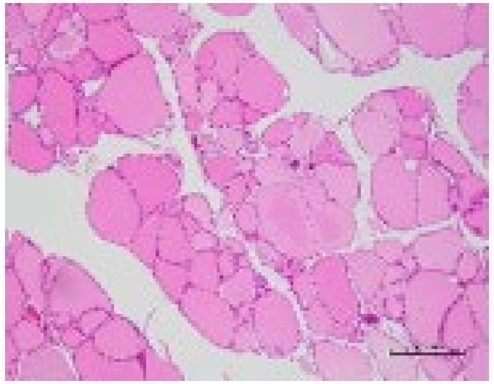
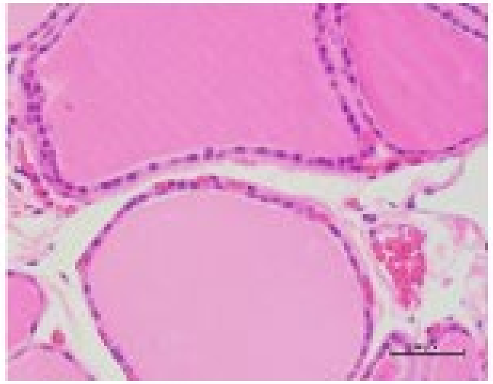
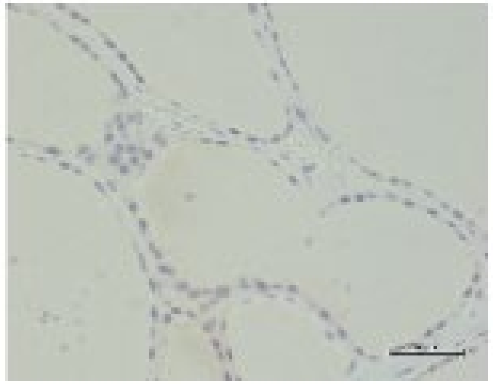
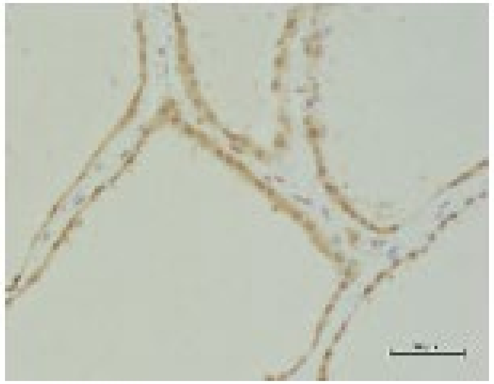
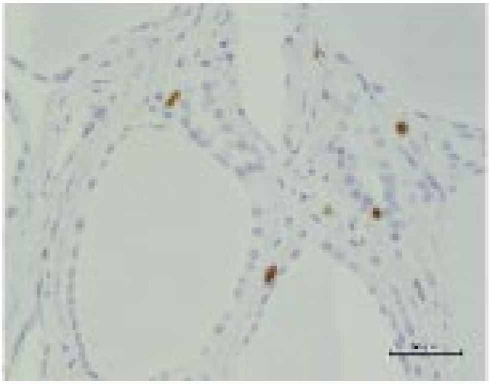

HE, low magnification

HE, high magnification

TLR2 score: 1x5=5

TLR3 score: 3x90=270

TLR4 score: 0

TLR5 score: 1x70+2x15=100

TLR7 score: 0

TLR9 score: 0

MyD88 score: 1x25=25

TRIF score: 1x40+2x15=70

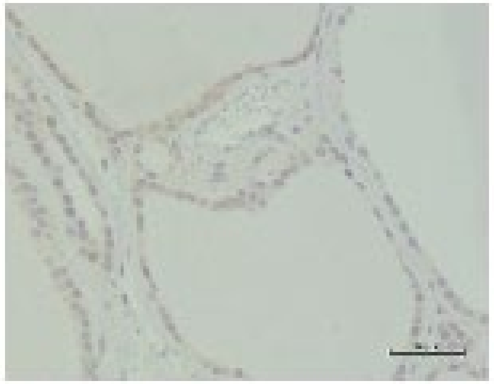
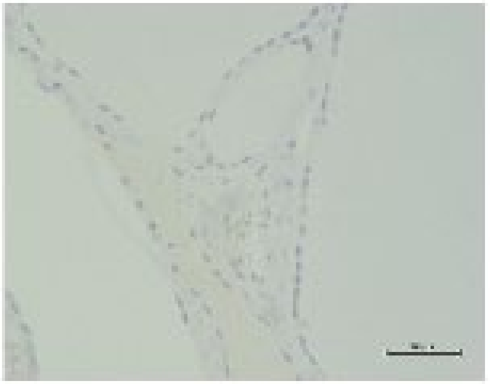
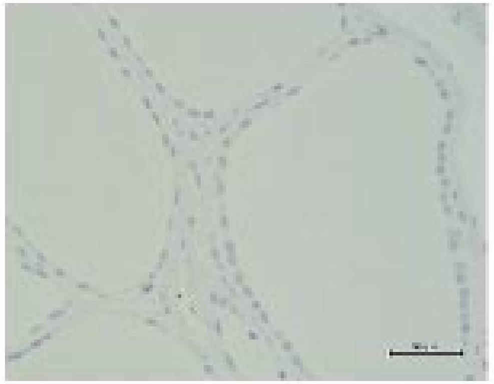
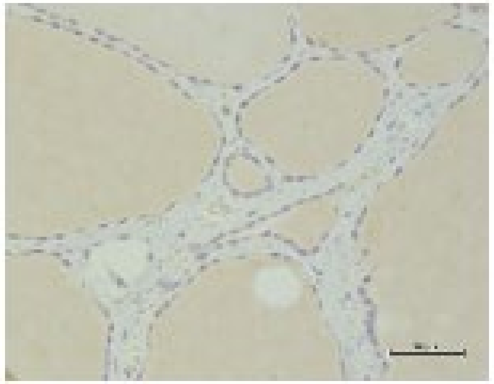
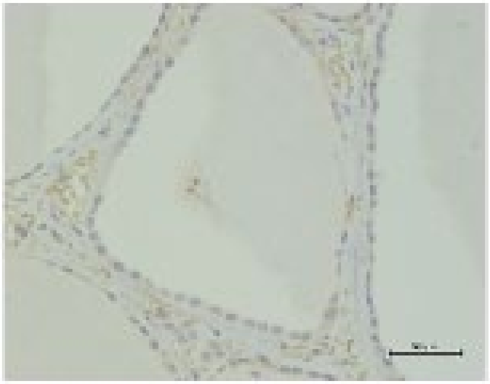

Figure S-1-4: HE and IHC images of NH, sample 4

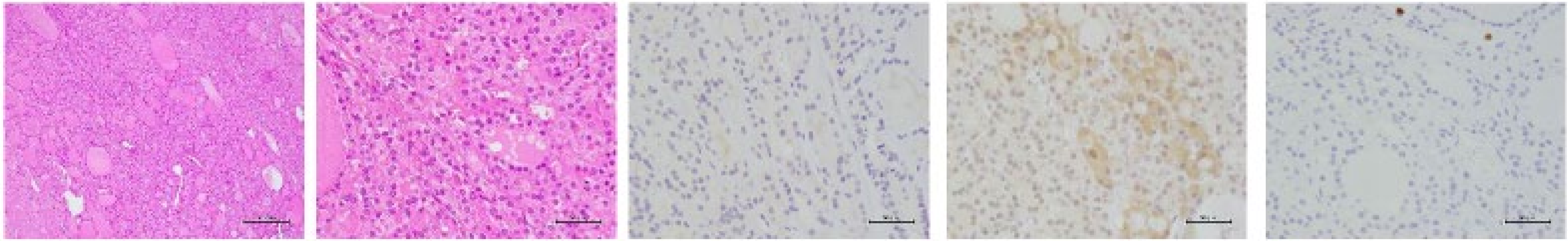

HE, low magnification

HE, high magnification

TLR2 score: 1x50=50

TLR3 score: 3x100=300

TLR4 score: 0

TLR5 score: 1x10=10

TLR7 score: 0

TLR9 score: 0

MyD88 score: 1x5=5

TRIF score: 1x40=40

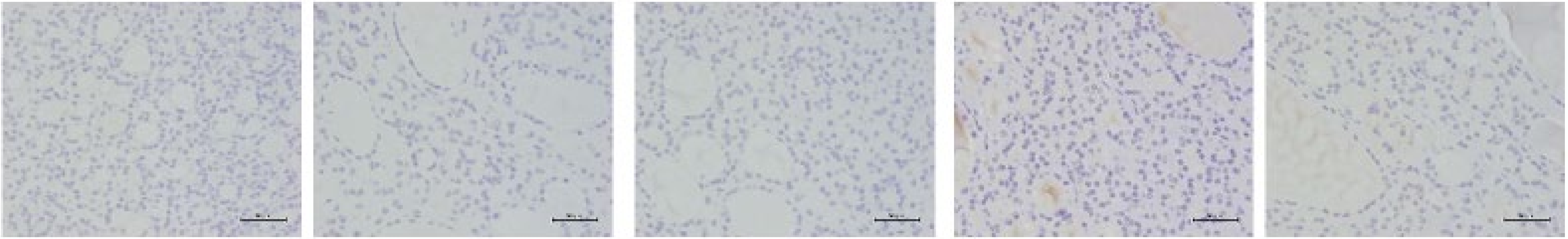

Figure S-1-5: HE and IHC images of NH, sample 5

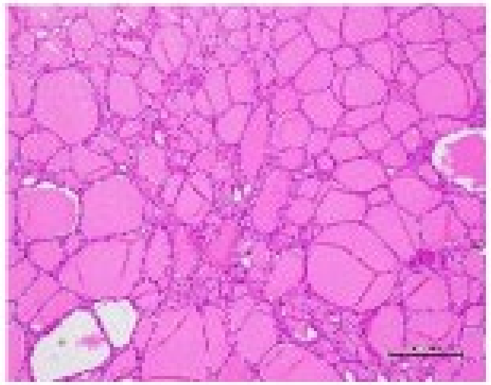

HE, low magnification

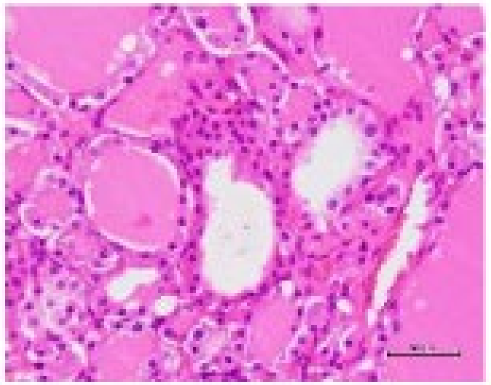

HE, high magnification

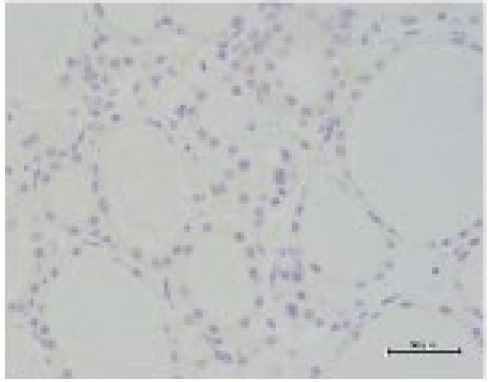

TLR2 score: 1x50=50

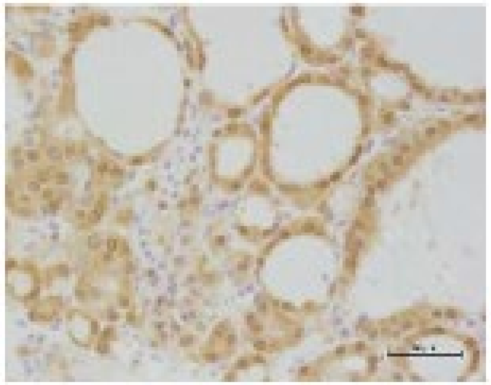

TLR3 score: 3x100=300

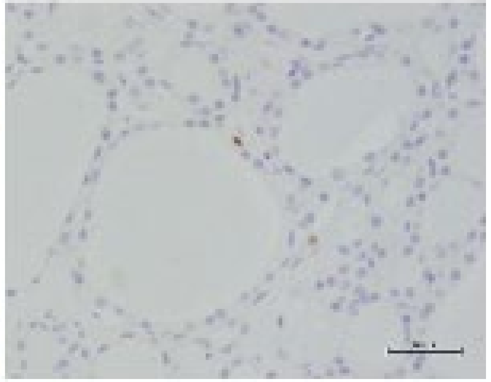

TLR4 score: 0

TLR5 score: 1x10+2x40+3x40=210

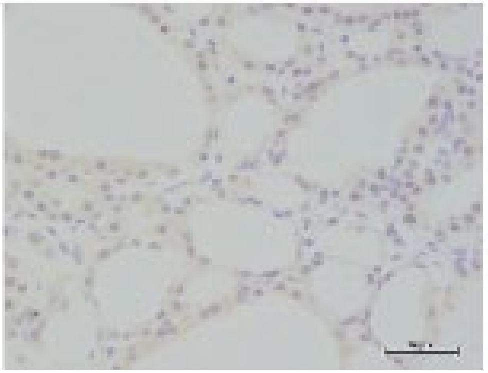

TLR7 score: 0

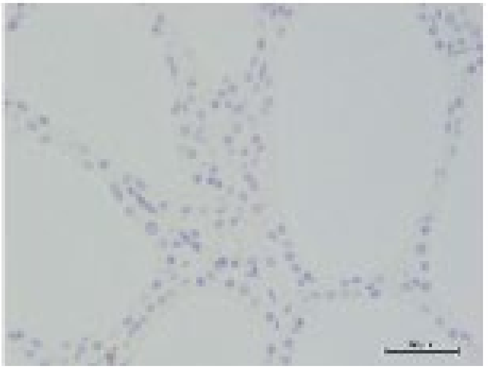

TLR9 score: 1x50=50

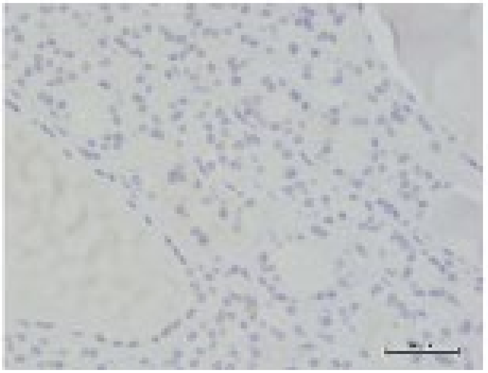

MyD88 score: 1x50=50

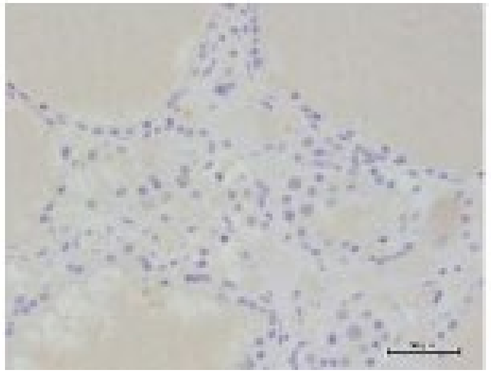

TRIF score: 1x75+2x20=115

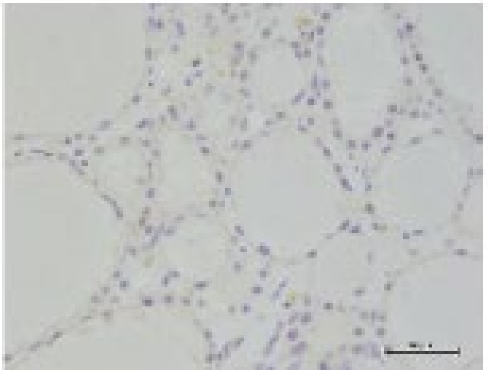

Figure S-1-6: HE and IHC images of NH, sample 6

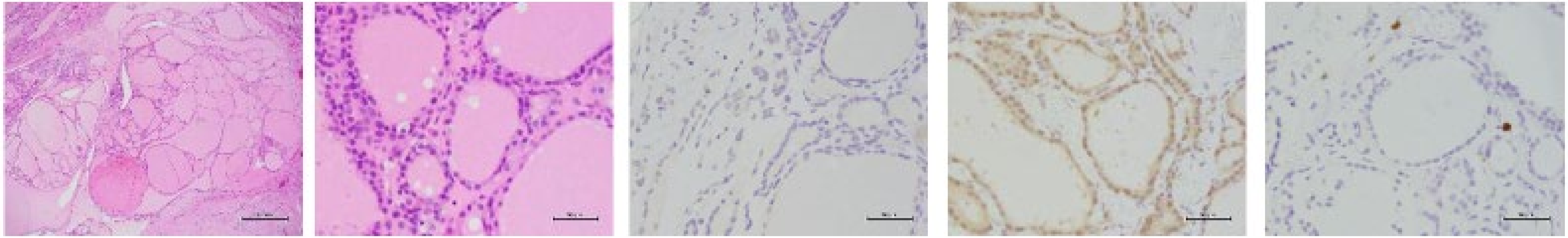

HE, low magnification

HE, high magnification

TLR2 score: 1x10=10

TLR3 score: 3x100=300

TLR4 score: 0

TLR5 score: 1x30+2x30+3x30=180

TLR7 score: 0

TLR9 score: 0

MyD88 score: 1x10+2x20=50

TRIF score: 1x30+2x15=60

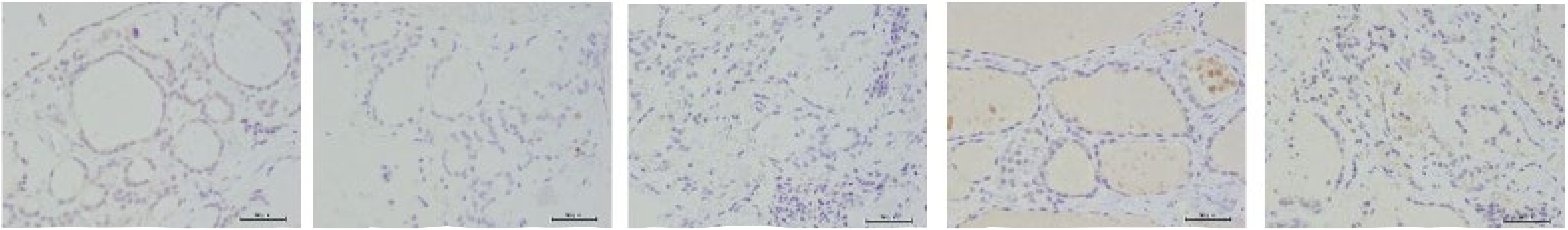

Figure S-1-7: HE and IHC images of NH, sample 7

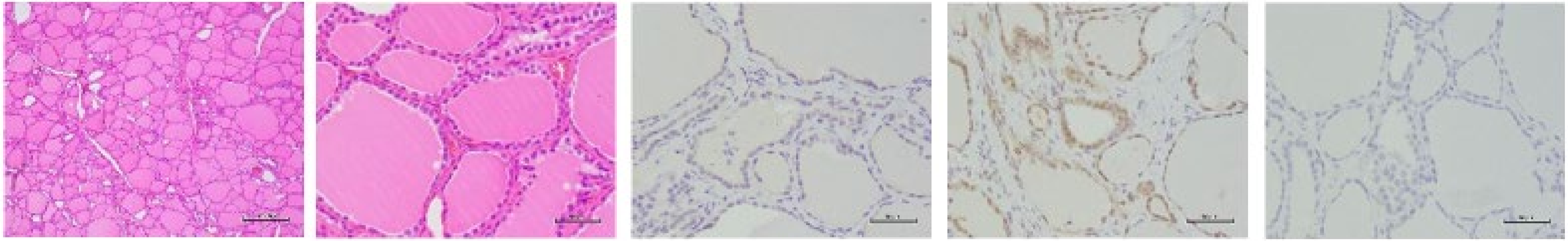

HE, low magnification

HE, high magnification

TLR2 score: 1x10=10

TLR3 score: 3x85=255

TLR4 score: 0

TLR5 score: 1x70=70

TLR7 score: 0

TLR9 score: 0

MyD88 score: 2x5+3x5=25

TRIF score: 1x40=40

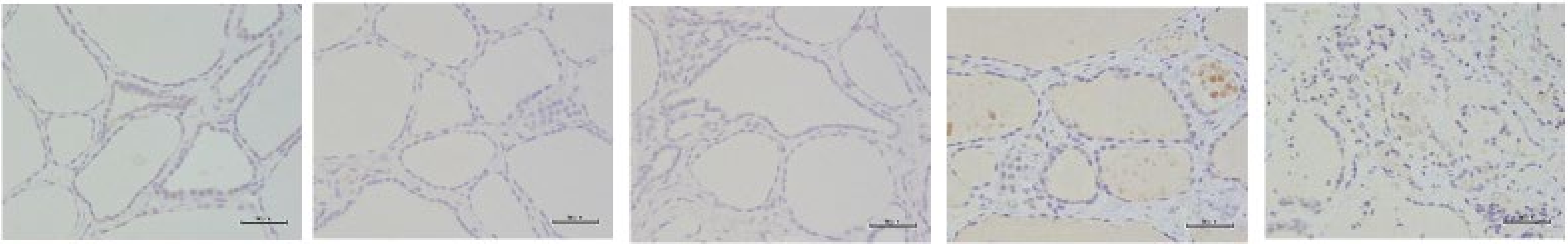

Figure S-1-8: HE and IHC images of NH, sample 8

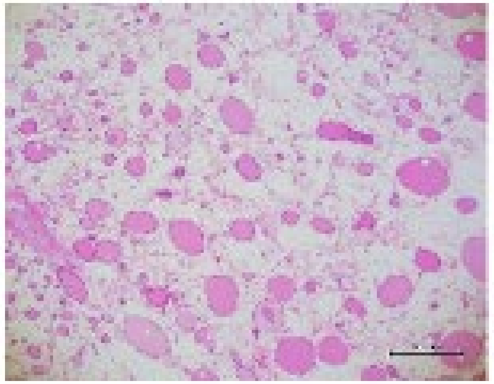
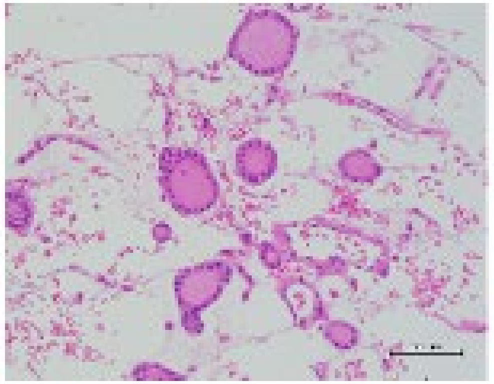
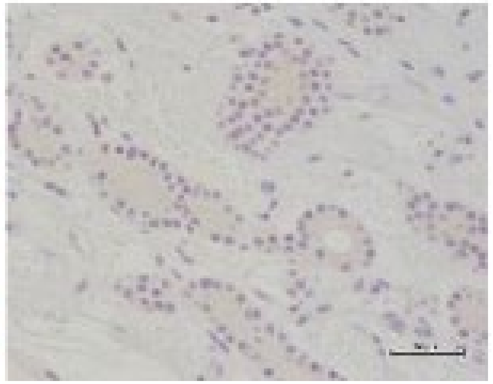
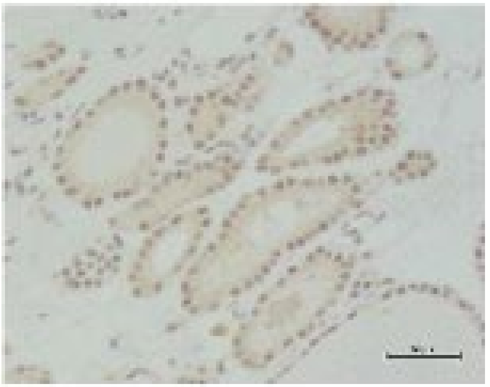
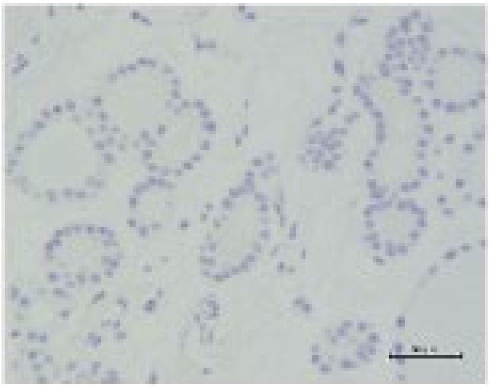

HE, low magnification

HE, high magnification

TLR2 score: 1x50+2x20=90

TLR3 score: 3x100=300

TLR4 score: 0

TLR5 score: 1x60=60

TLR7 score: 0

TLR9 score: 0

MyD88 score: 1x10=10

TRIF score: 1x50+2x20=90

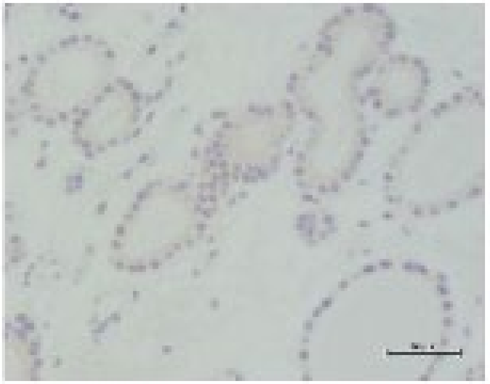
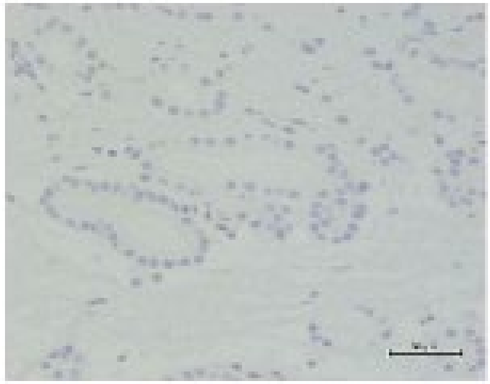
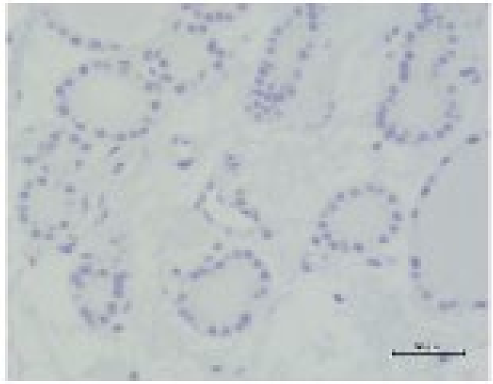
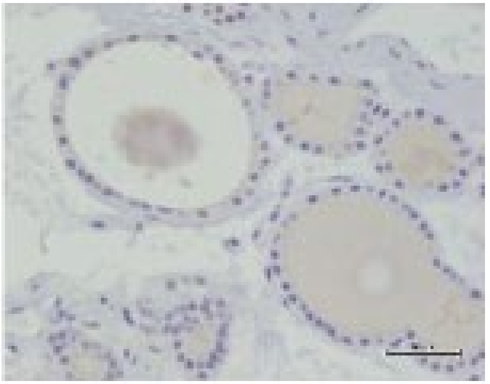
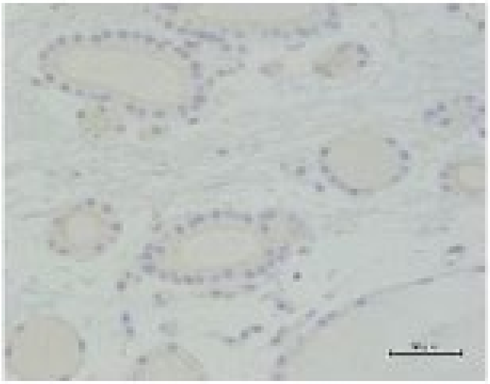

Figure S-2-1: HE and IHC images of PTC, sample 1

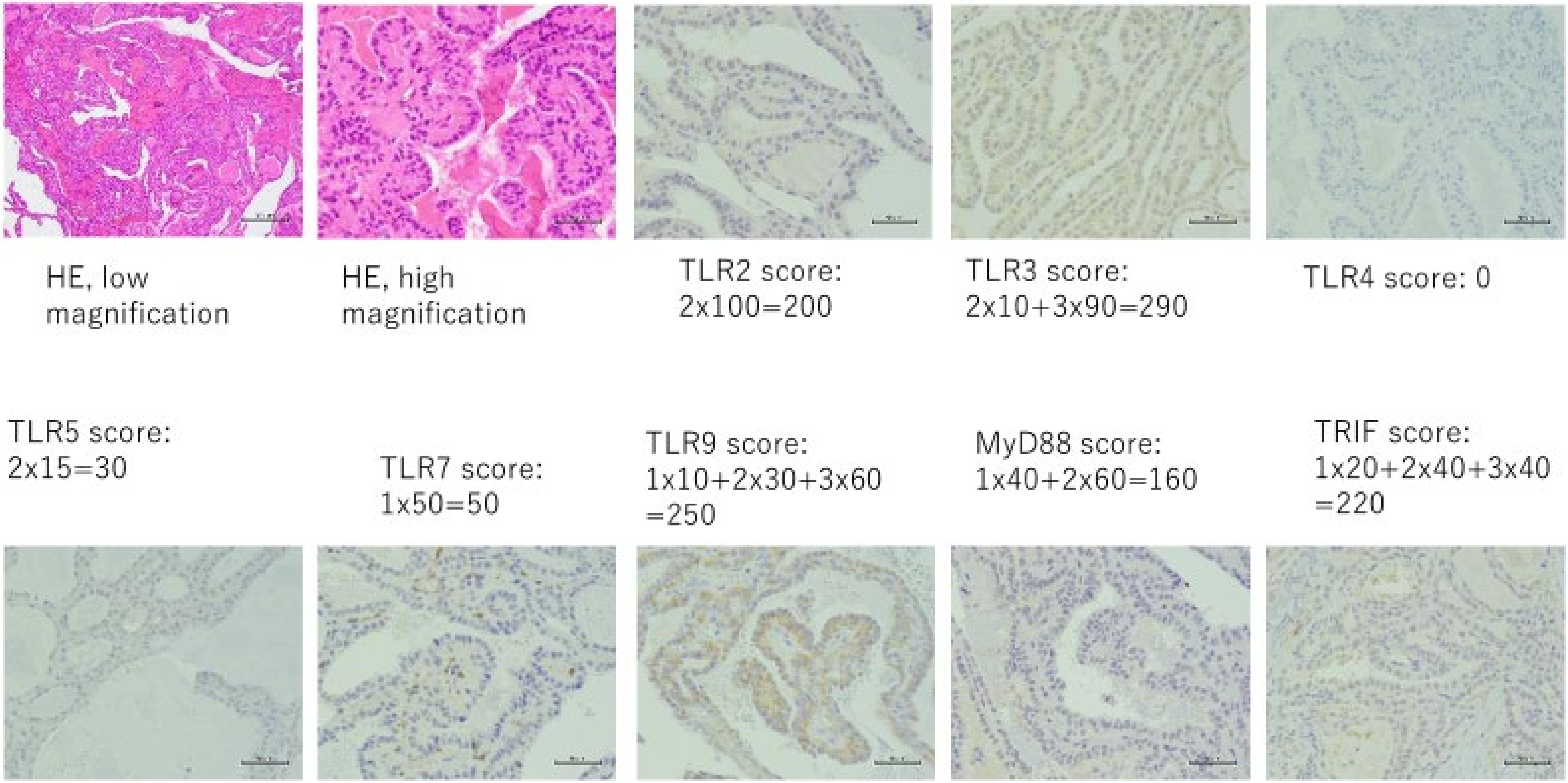

Figure S-2-2: HE and IHC images of PTC, sample 2

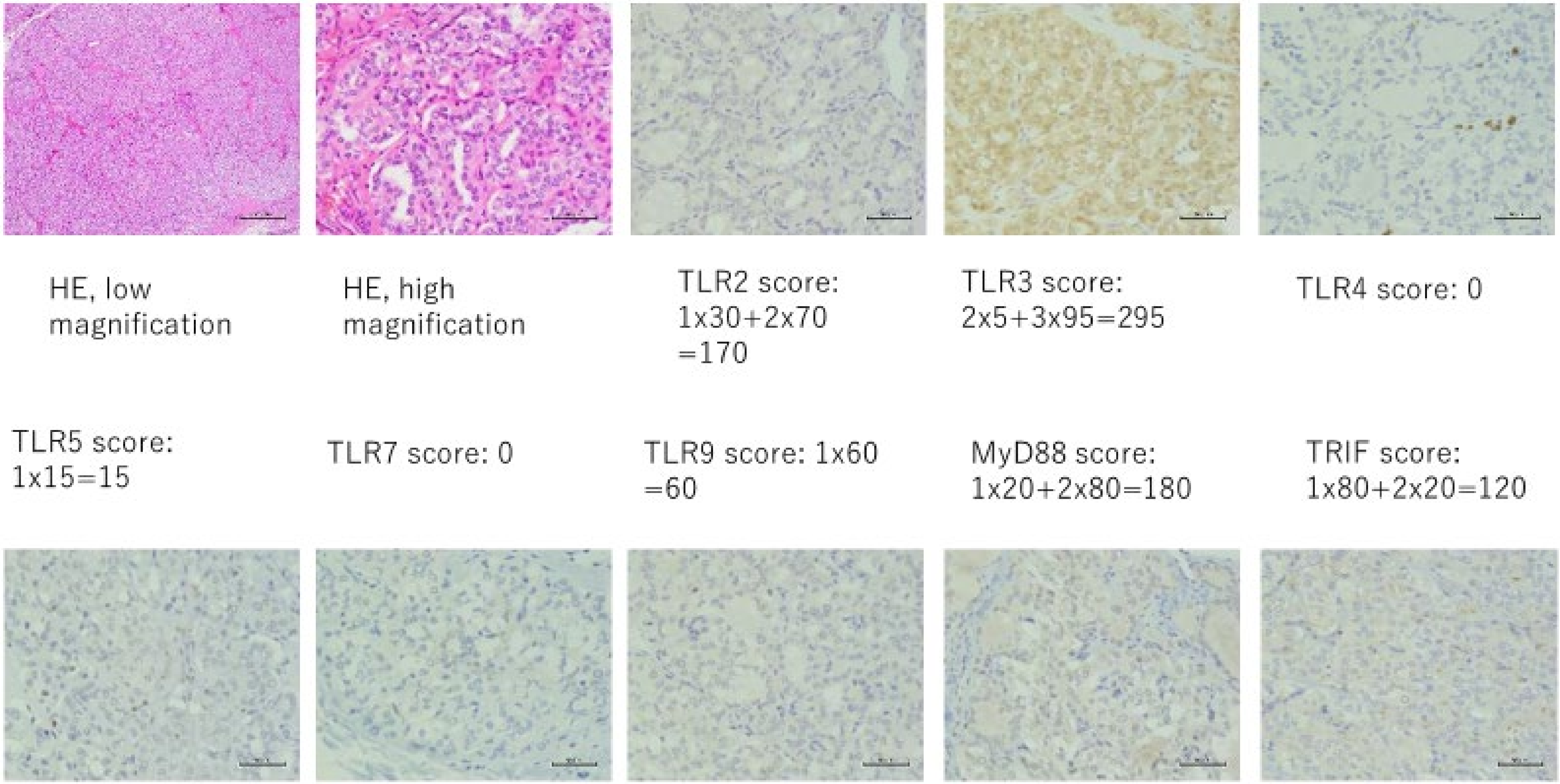

Figure S-2-3: HE and IHC images of PTC, sample 3

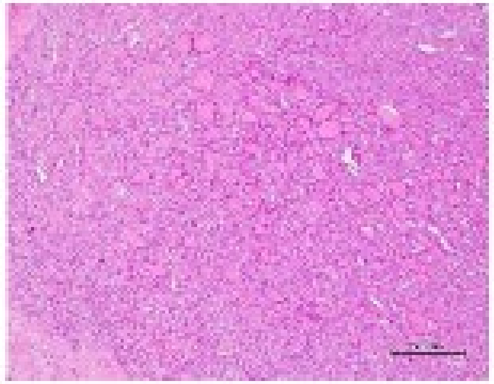
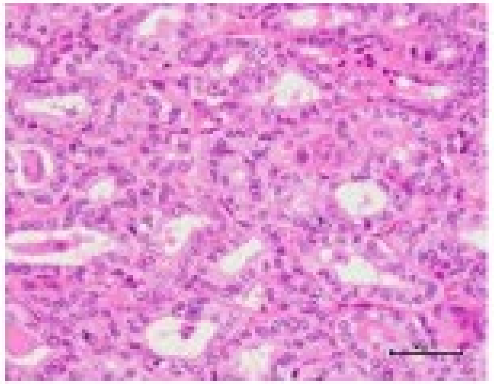
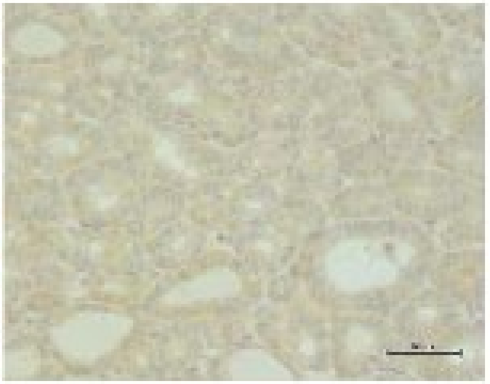
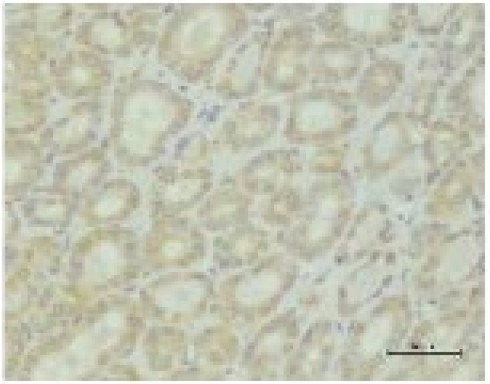
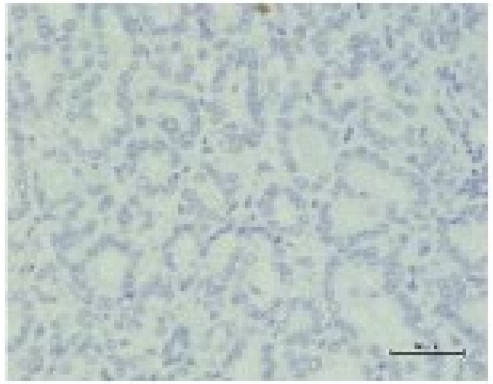

HE, low magnification

HE, high magnification

TLR2 score: 3x100=300

TLR3 score: 2x5+3x95=295

TLR4 score: 0

TLR5 score: 1x5=5

TLR7 score: 0

TLR9 score: 1x80=80

MyD88 score: 1x40+2x60=160

TRIF score: 1x50=50

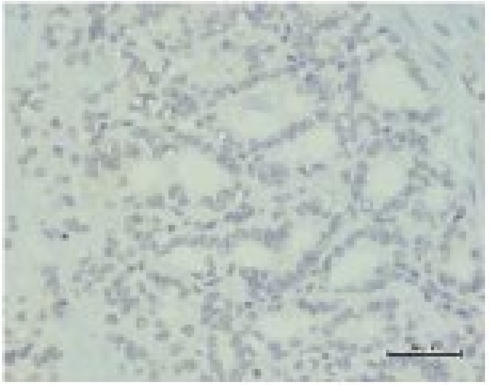
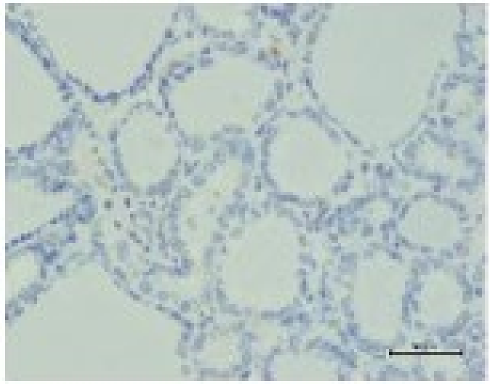
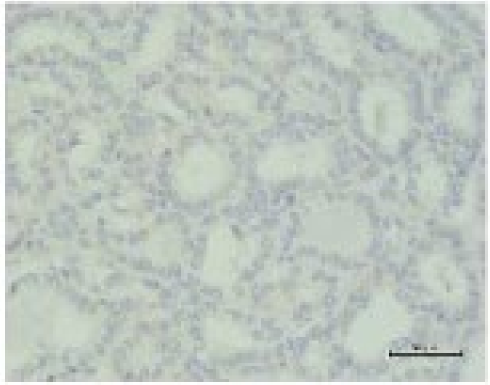
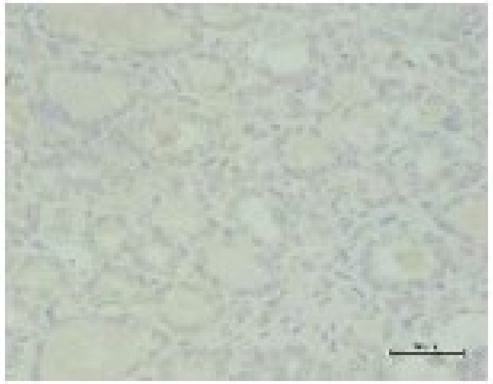
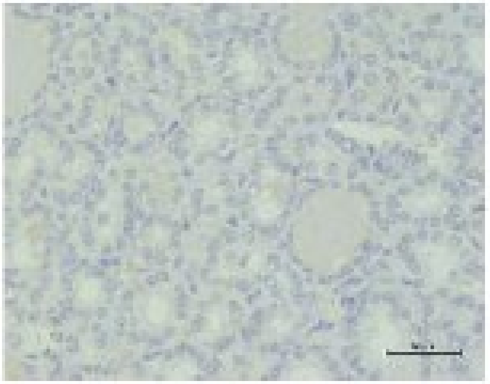

Figure S-2-4: HE and IHC images of PTC, sample 4

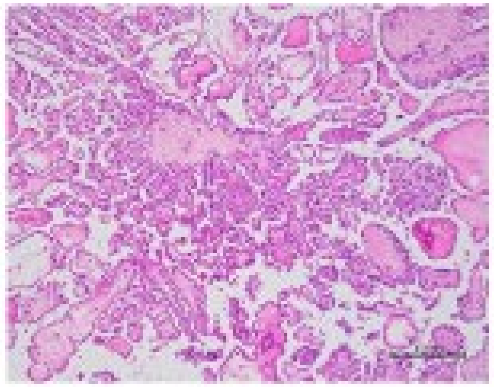

HE, low magnification

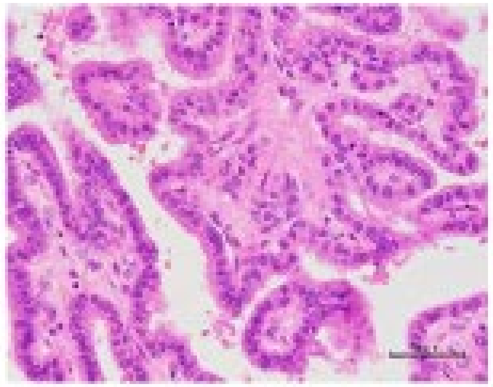

HE, high magnification

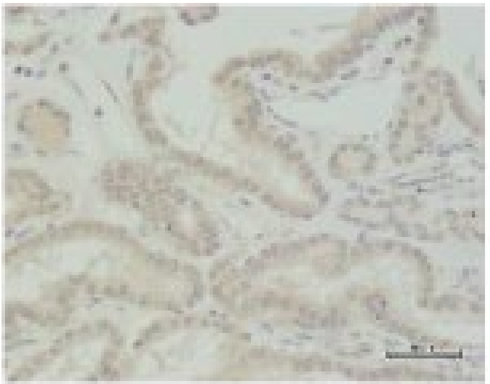

TLR2 score: 3x100=300

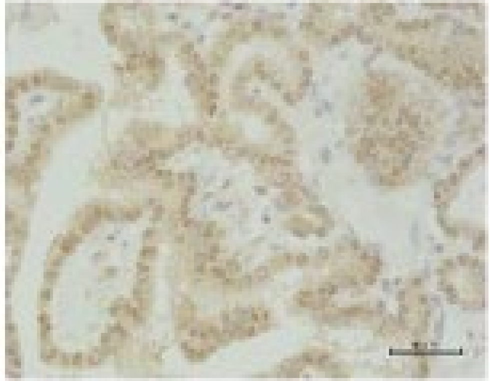

TLR3 score: 3x100=300

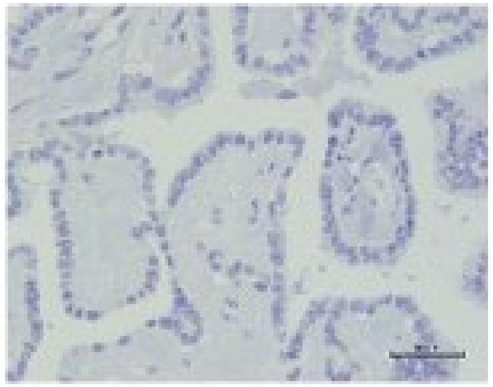

TLR4 score: 0

TLR5 score: 1x5=5

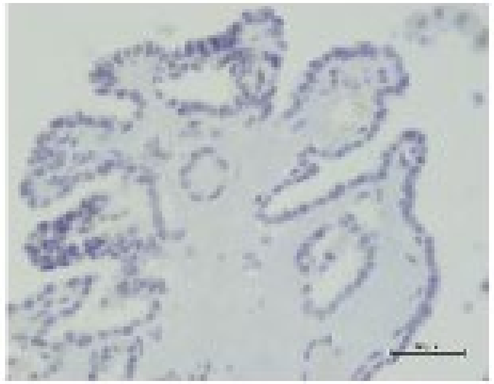

TLR7 score: 0

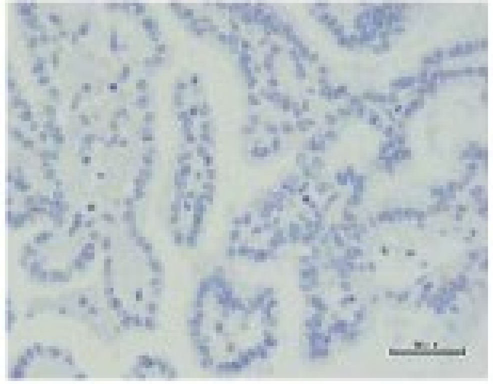

TLR9 score: 1x30+2x40+3x30 =200

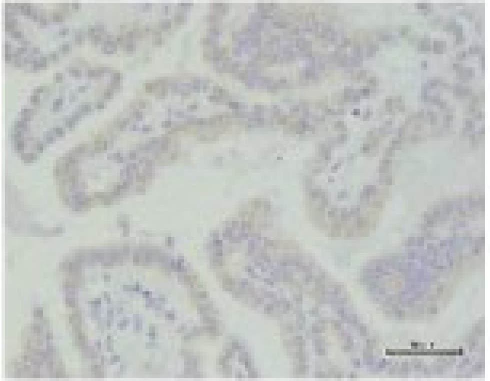

MyD88 score: 1x50+2x5=60

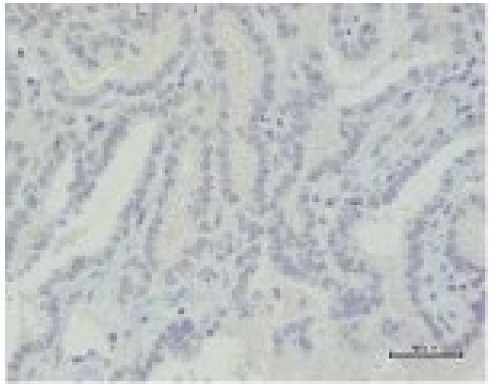

TRIF score: 1x60=60

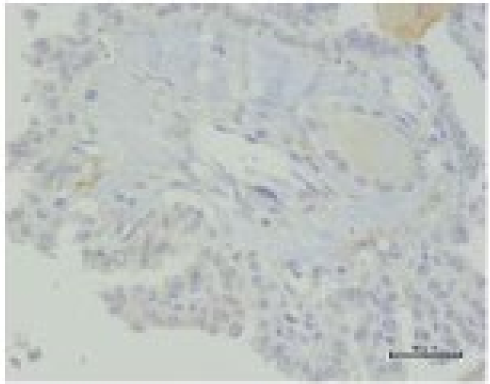

Figure S-2-5: HE and IHC images of PTC, sample 5

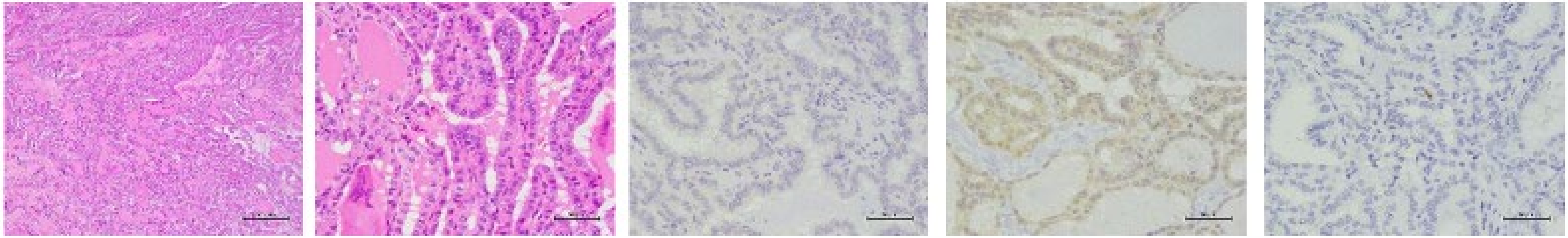

HE, low magnification

HE, high magnification

TLR2 score: 1x65=65

TLR3 score: 3x100=300

TLR4 score: 0

TLR5 score: 1x5=5

TLR7 score: 0

TLR9 score: 1x30+2x60=150

MyD88 score: 2x100=200

TRIF score: 1x70=70

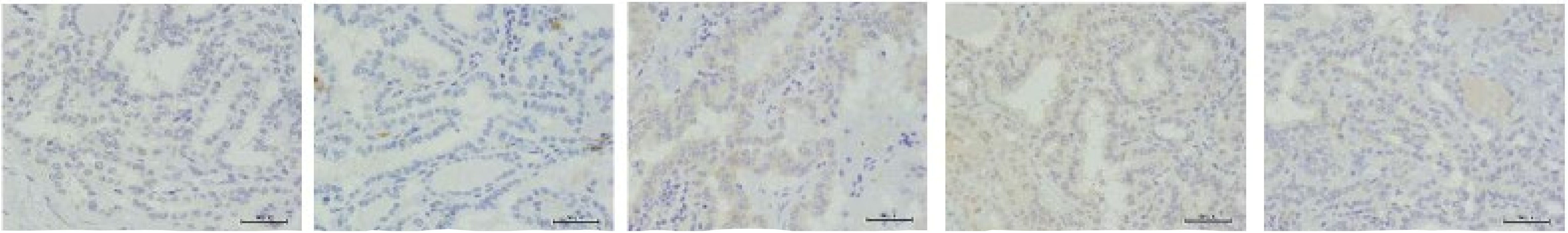

Figure S-2-6: HE and IHC images of PTC, sample 6

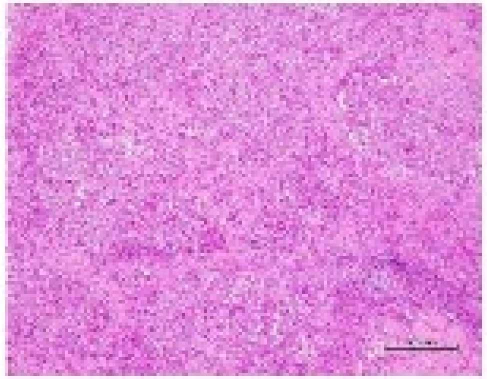

HE, low magnification

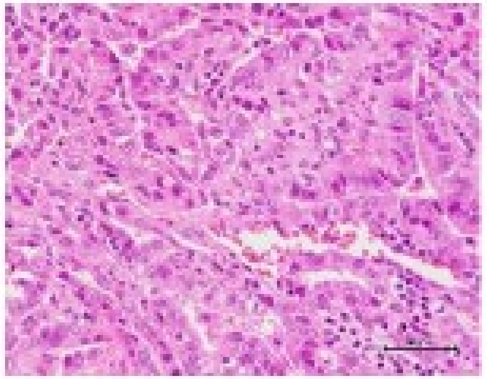

HE, high magnification

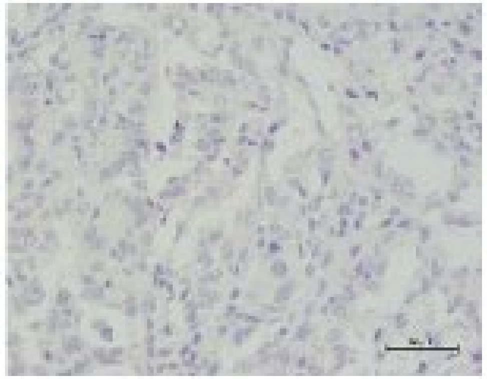

TLR2 score: 1x90=90

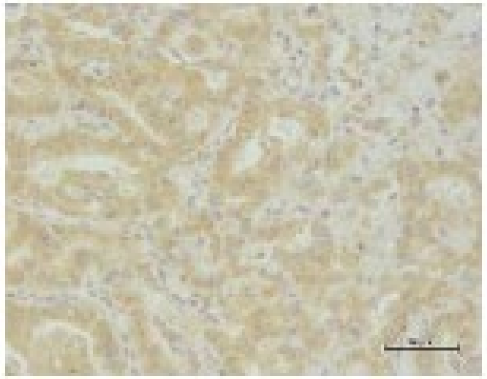

TLR3 score: 3x100=300

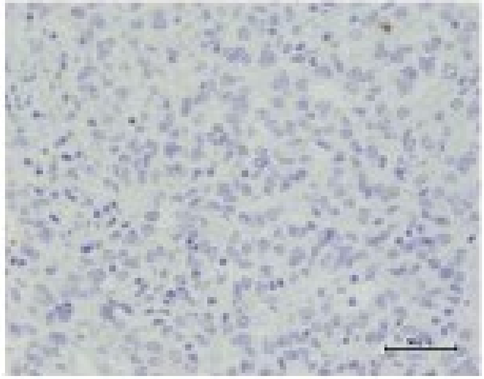

TLR4 score: 0

TLR5 score: 1x30=30

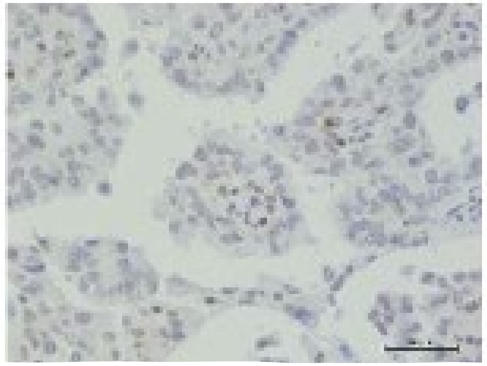

TLR7 score: 0

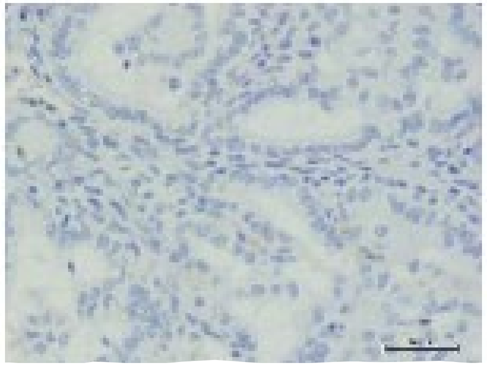

TLR9 score: 1x10+2x5+3x80 =260

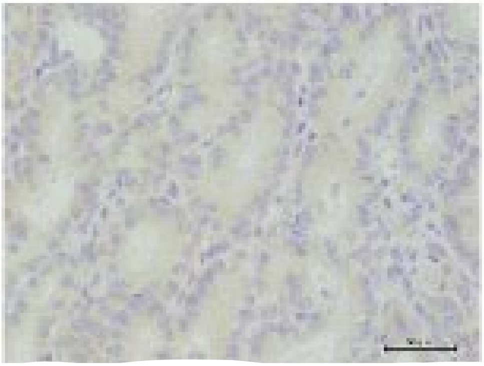

MyD88 score: 1x35+2x60+3x5 =170

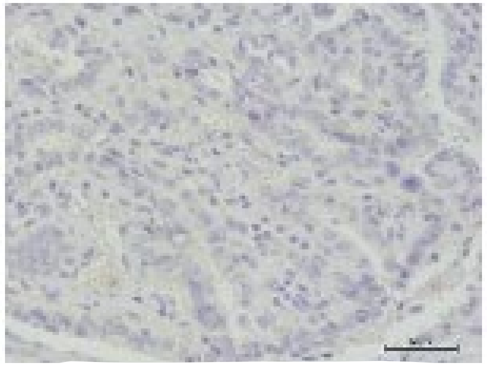

TRIF score: 1x55+2x35=125

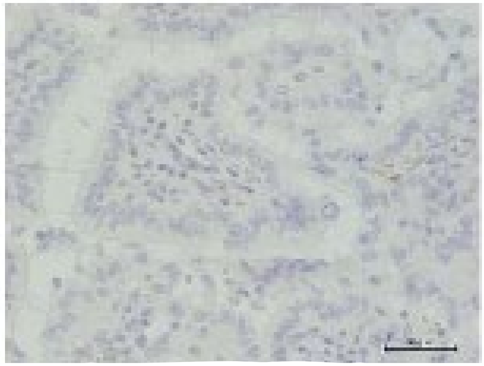

Figure S-2-7: HE and IHC images of PTC, sample 7

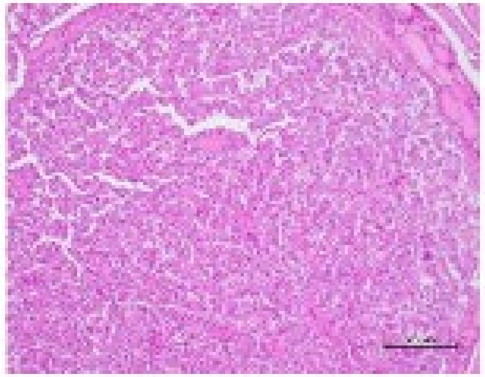

HE, low magnification

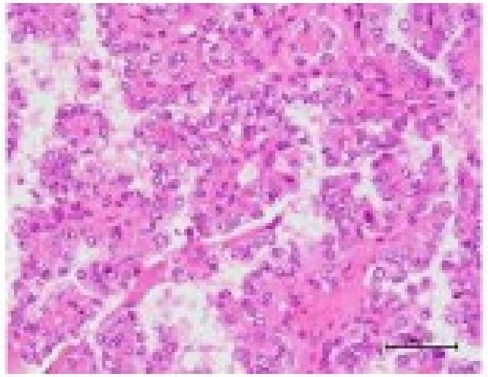

HE, high magnification

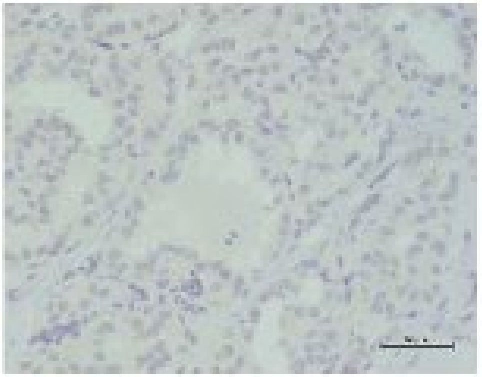

TLR2 score: 2x100=200

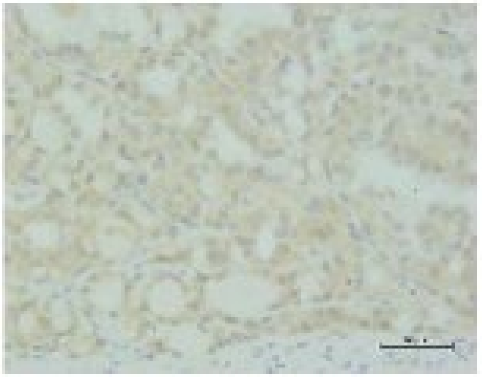

TLR3 score: 2x30+3x70=270

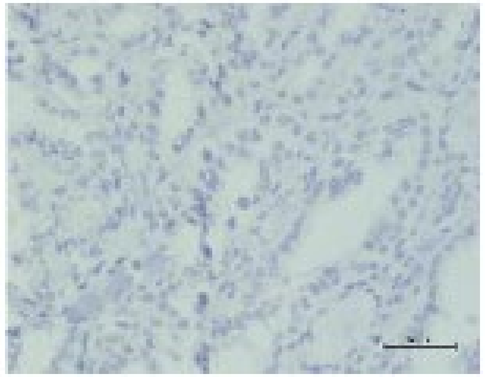

TLR4 score: 0

TLR5 score: 1x5=5

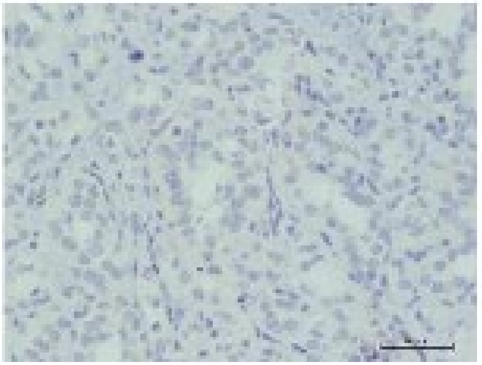

TLR7 score: 0

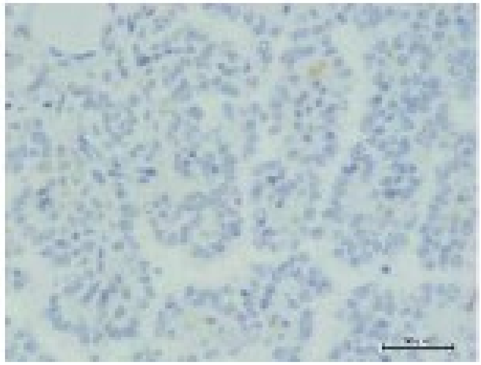

TLR9 score: 1x40+2x40+3x10 =150

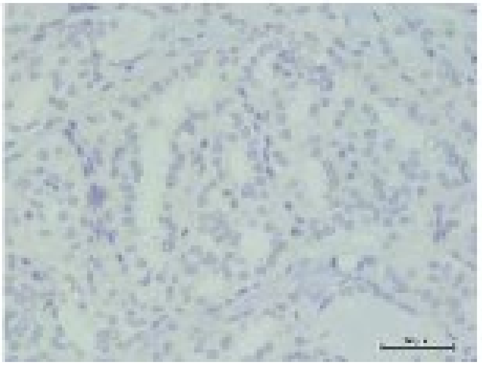

MyD88 score: 1x20+2x55+3x25 =250

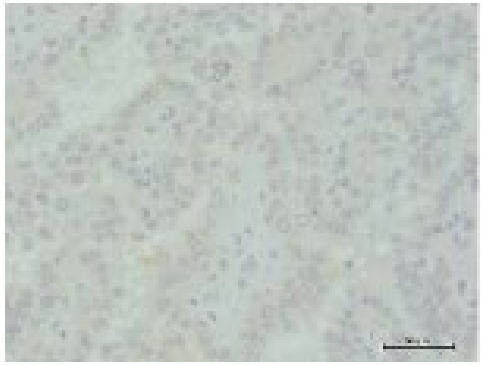

TRIF score: 1x40+2x60=160

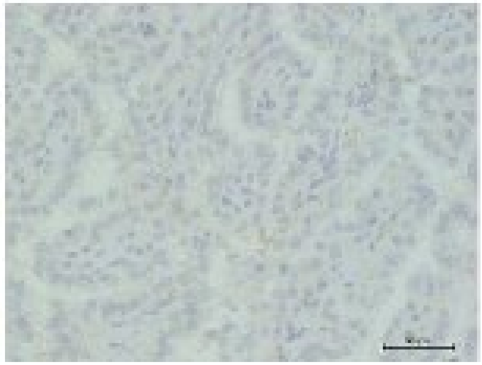

Figure S-2-8: HE and IHC images of PTC, sample 8

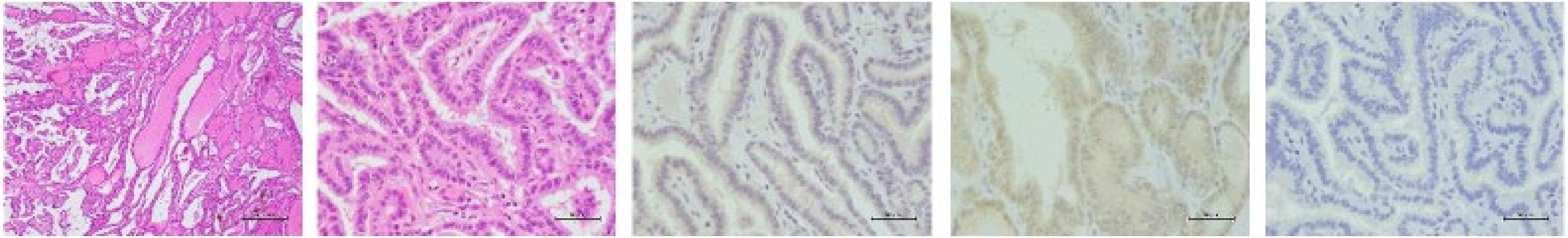

HE, low magnification

HE, high magnification

TLR2 score: 2x30+3x70=270

TLR3 score: 2x10+3x90=290

TLR4 score: 0

TLR5 score: 1x3=3

TLR7 score: 1x2=2

TLR9 score: 1x20+2x10=40

MyD88 score: 1x40=40

TRIF score: 1x55+2x45=145

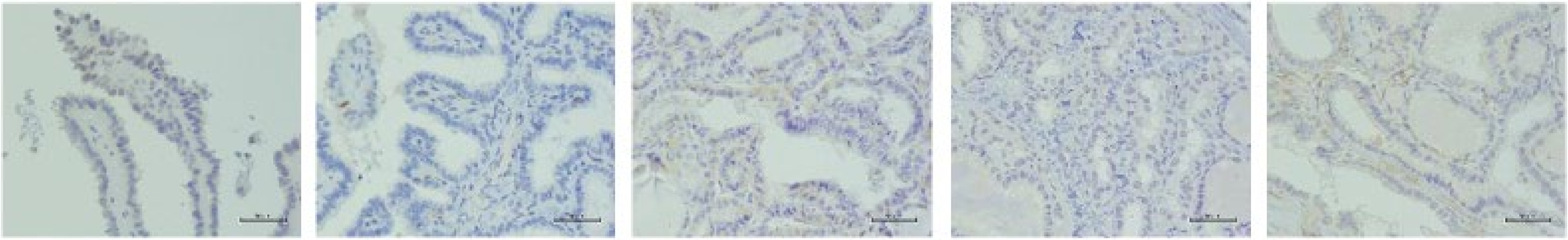

Figure S-2-9: HE and IHC images of PTC, sample 9

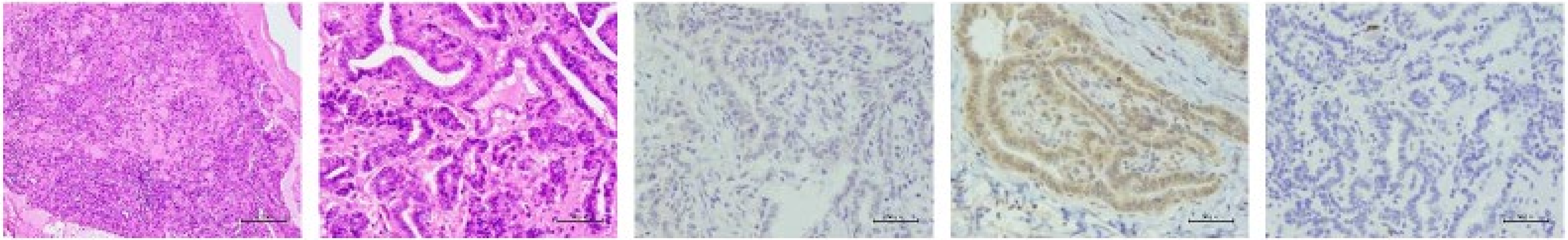

HE, low magnification

HE, high magnification

TLR2 score: 1x70+2x20 =110

TLR3 score: 2x10+3x90=290

TLR4 score: 0

TLR5 score: 1x5+2x2=9

TLR7 score: 0

TLR9 score: 1x50+2x10=70

MyD88 score: 1x25=25

TRIF score: 1x30+2x40 +3x30=200

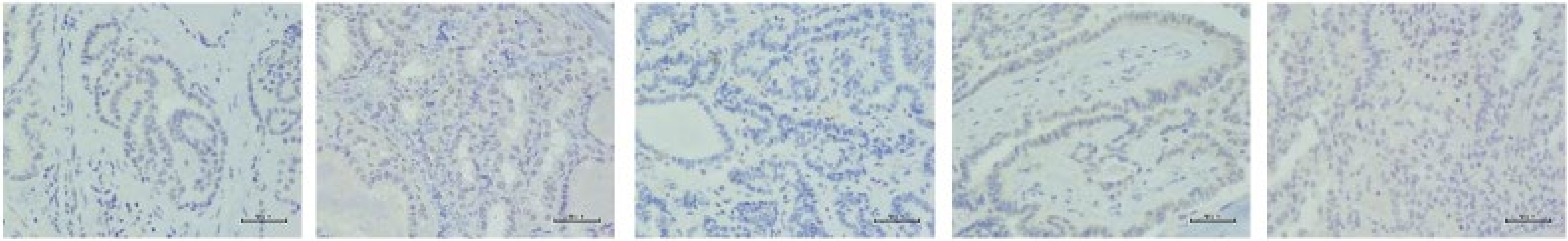

Figure S-2-10: HE and IHC images of PTC, sample 10

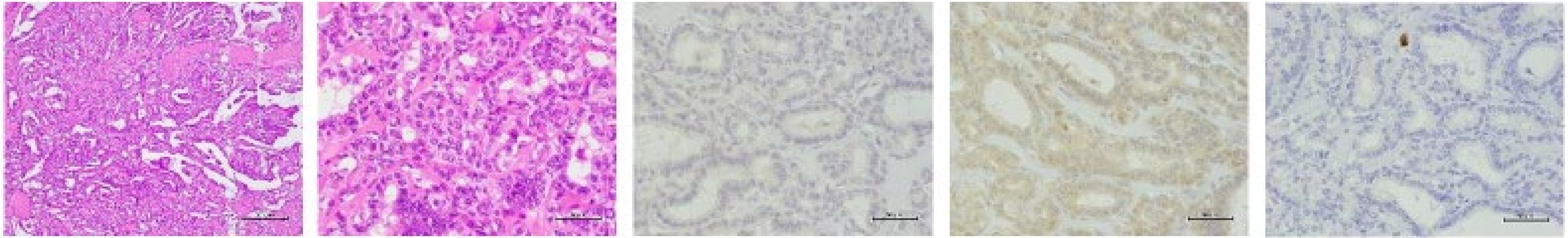

HE, low magnification

HE, high magnification

TLR2 score: 2x80＋3 x 20 =220

TLR3 score: 3x100=300

TLR4 score: 0

TLR5 score: 0

TLR7 score: 0

TLR9 score: 0

MyD88 score: 2x100=200

TRIF score: 1x100=100

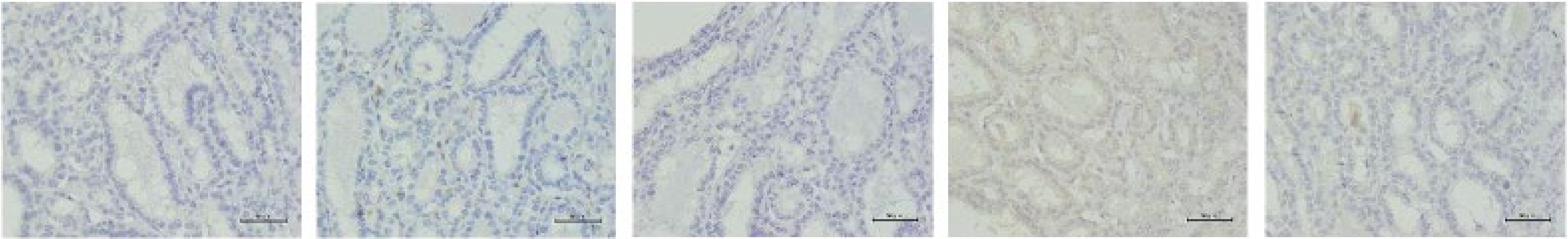

Figure S-2-11: HE and IHC images of PTC, sample 11

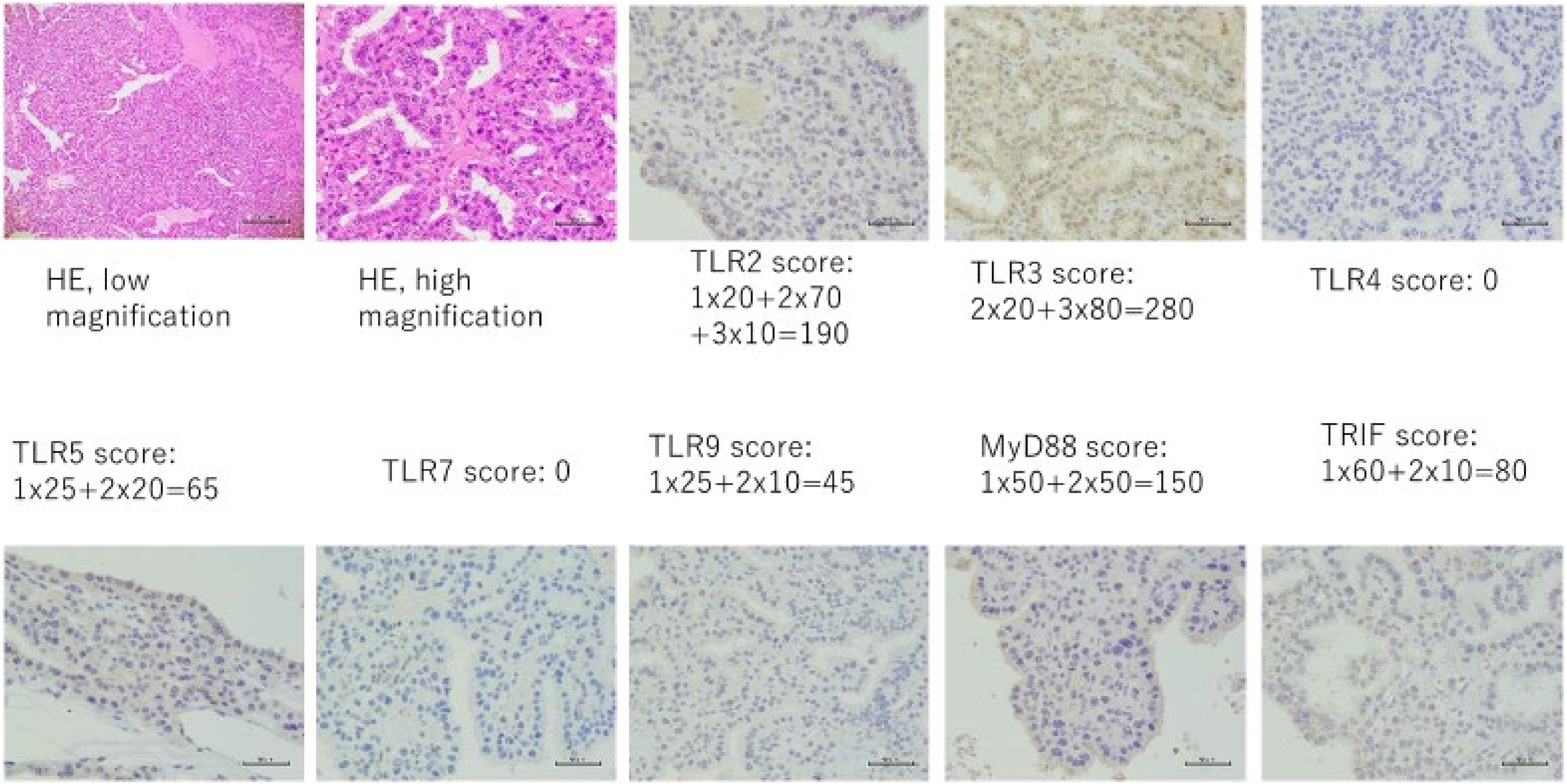

Figure S-2-12: HE and IHC images of PTC, sample 11

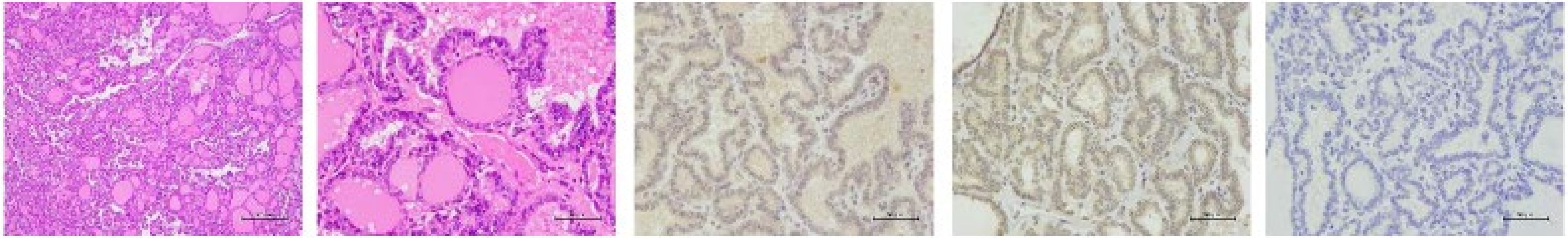

HE, low magnification

HE, high magnification

TLR2 score: 3x100=300

TLR3 score: 2x20+3x80=280

TLR4 score: 0

TLR5 score: 1x10=10

TLR7 score: 0

TLR9 score: 1x10+2x5=20

MyD88 score: 1x30+2x70=170

TRIF score: 1x10=10

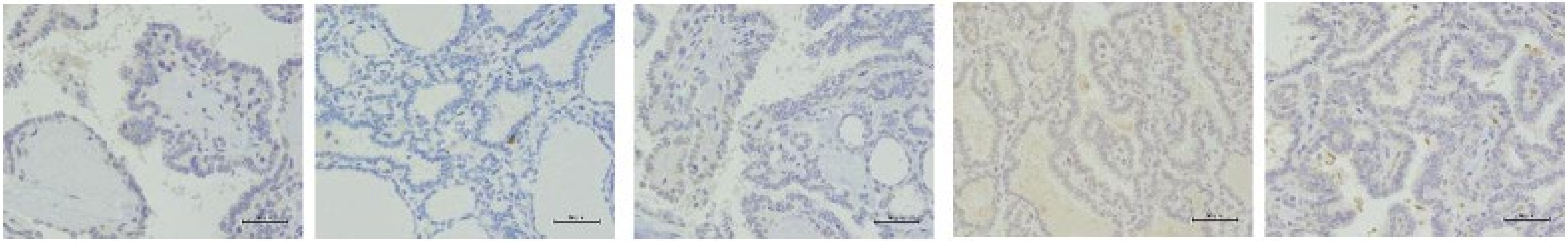

Figure S-2-13: HE and IHC images of PTC, sample 13

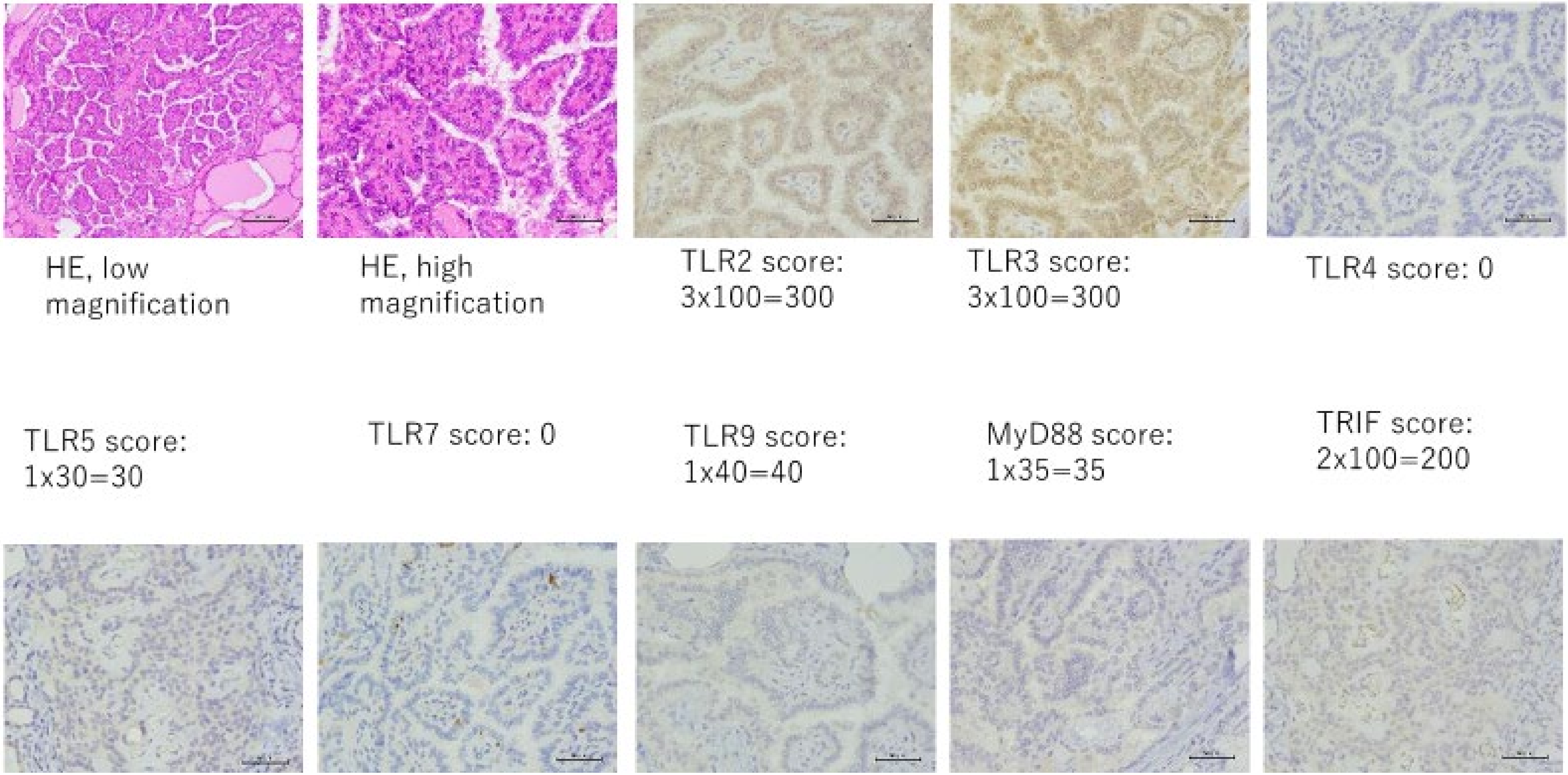

Figure S-2-14: HE and IHC images of PTC, sample 14

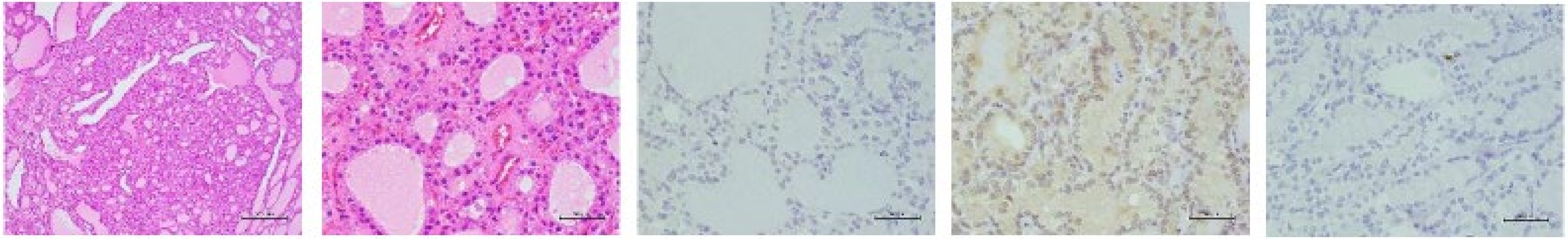

HE, low magnification

HE, high magnification

TLR2 score: 1x100=100

TLR3 score: 3x100=300

TLR4 score: 0

TLR5 score: 0

TLR7 score: 0

TLR9 score: 0

MyD88 score: 2x100=200

TRIF score: 1x100=100

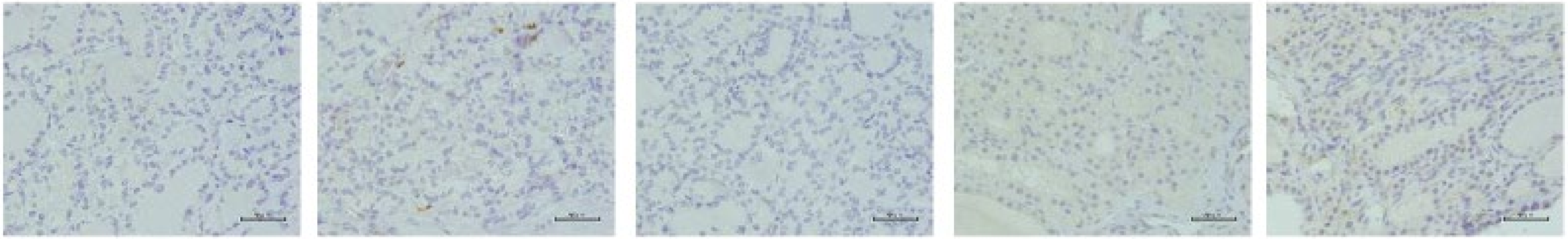

Figure S-2-15: HE and IHC images of PTC, sample 15

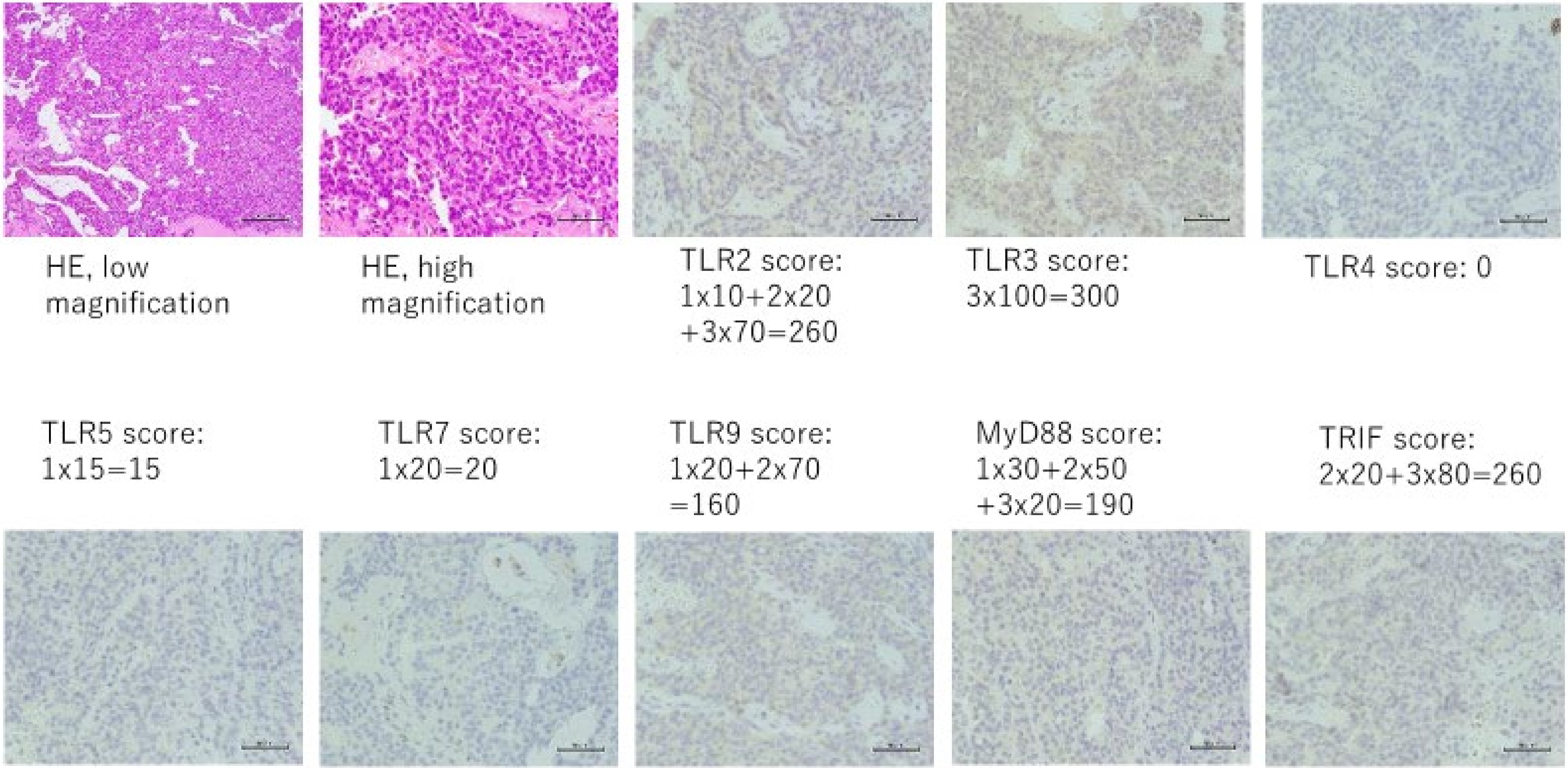

Figure S-2-16: HE and IHC images of PTC, sample 16

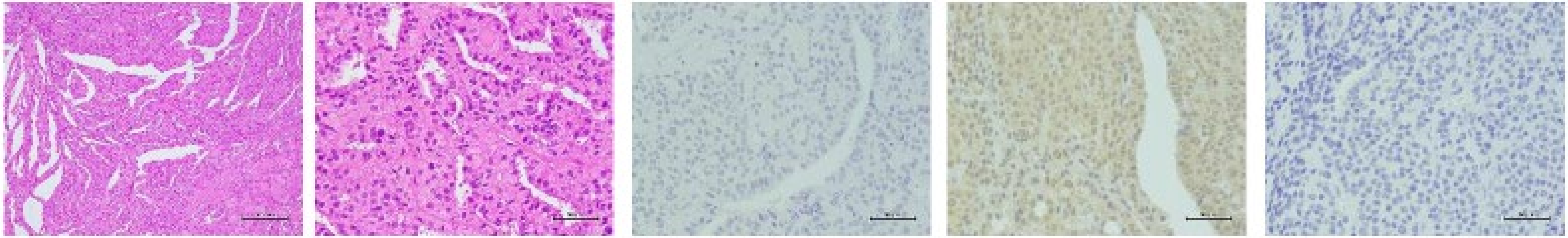

HE, low magnification

HE, high magnification

TLR2 score: 1x65=65

TLR3 score: 3x100=300

TLR4 score: 0

TLR5 score: 1x75=75

TLR7 score: 1x5=5

TLR9 score: 1x40+2x20+3x40 =200

MyD88 score: 1x50+2x40 +3x10=160

TRIF score: 2x40+3x60=260

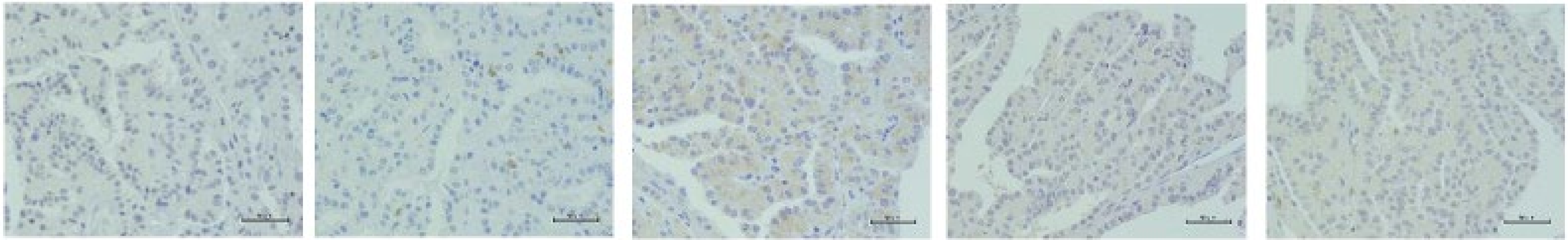

Figure S-2-17: HE and IHC images of PTC, sample 17

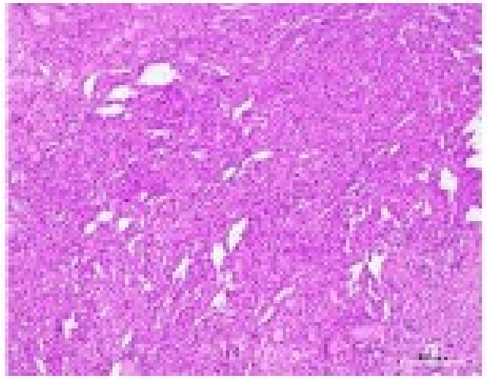

HE, low magnification

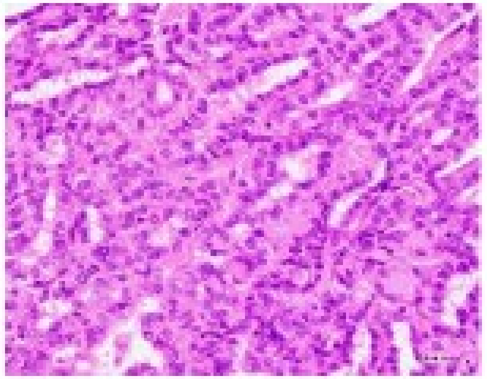

HE, high magnification

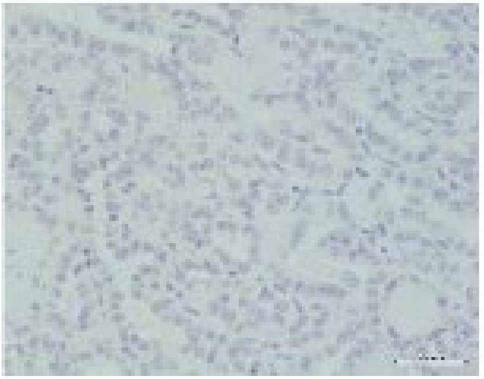

TLR2 score: 1x100=200

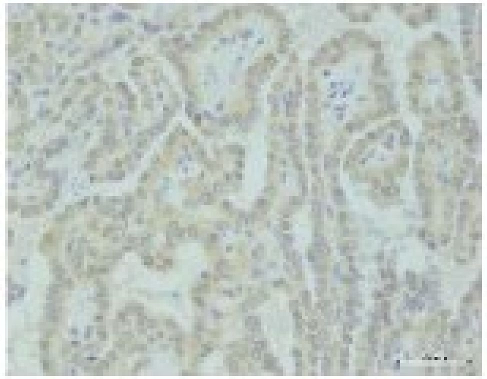

TLR3 score: 3x100=300

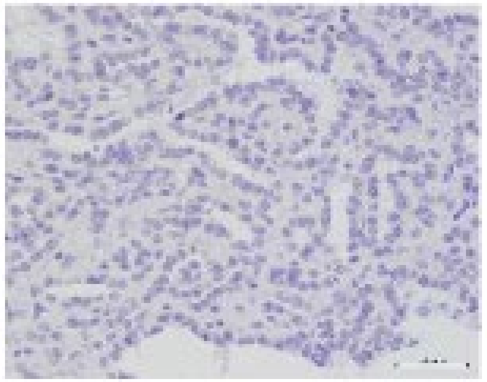

TLR4 score: 0

TLR5 score: 0

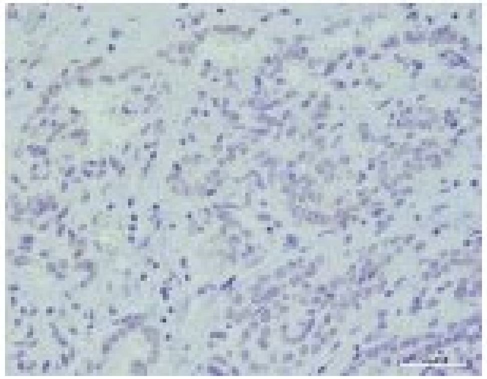

TLR7 score: 1x40+2x10=60

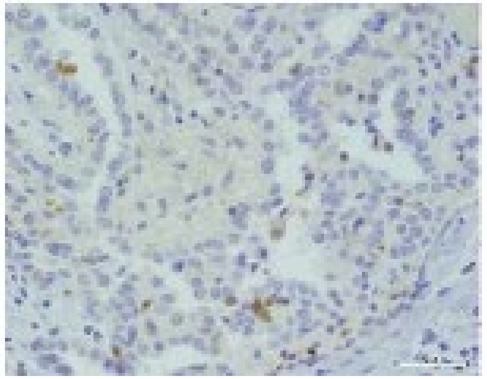

TLR9 score: 2x10+3x80=260

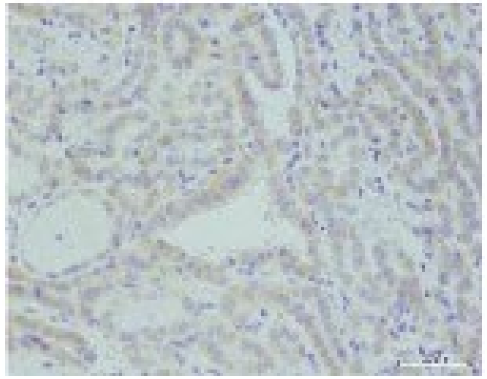

MyD88 score: 1x10=10

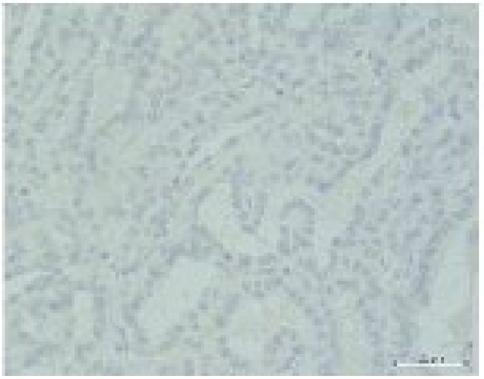

TRIF score: 1x50+2x50=150

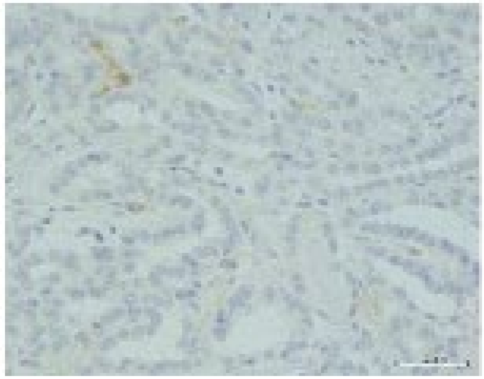

Figure S-2-18: HE and IHC images of PTC, sample 18

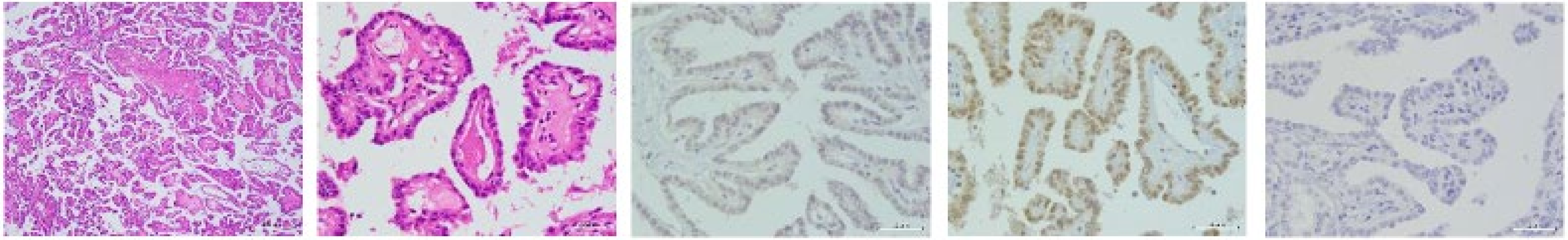

HE, low magnification

HE, high magnification

TLR2 score: 3x85=255

TLR3 score: 2x20+3x80=280

TLR4 score: 0

TLR5 score: 1x50=50

TLR7 score: 1x15+2x5=25

TLR9 score: 1x10+2x20 +3x60=200

MyD88 score: 1x50=50

TRIF score: 1x50+2x50=150

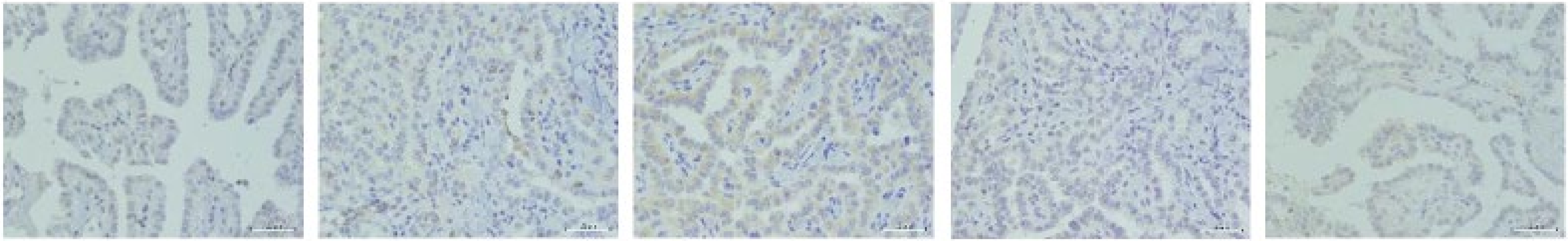

Figure S-2-19: HE and IHC images of PTC, sample 19

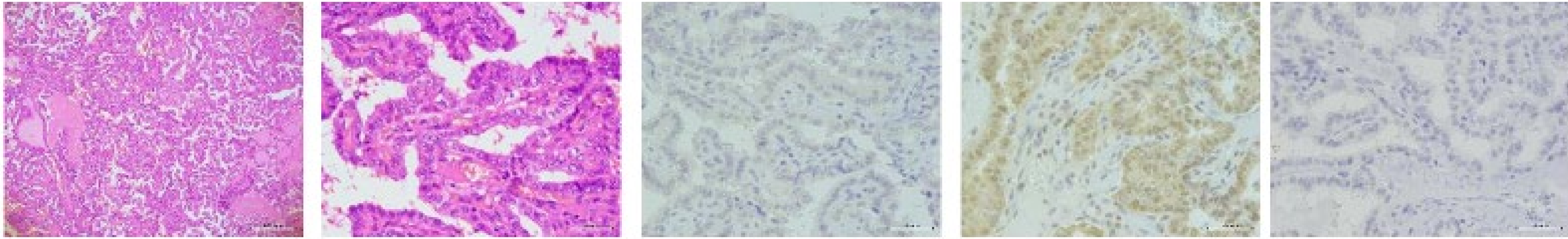

HE, low magnification

HE, high magnification

TLR2 score: 2x50+3x50 =250

TLR3 score: 3x100=300

TLR4 score: 0

TLR5 score: 1x5=5

TLR7 score: 1x30+2x10 +3x20=110

TLR9 score: 1x10+2x40+3x40 =230

MyD88 score: 3x100=300

TRIF score: 2x20+3x80=260

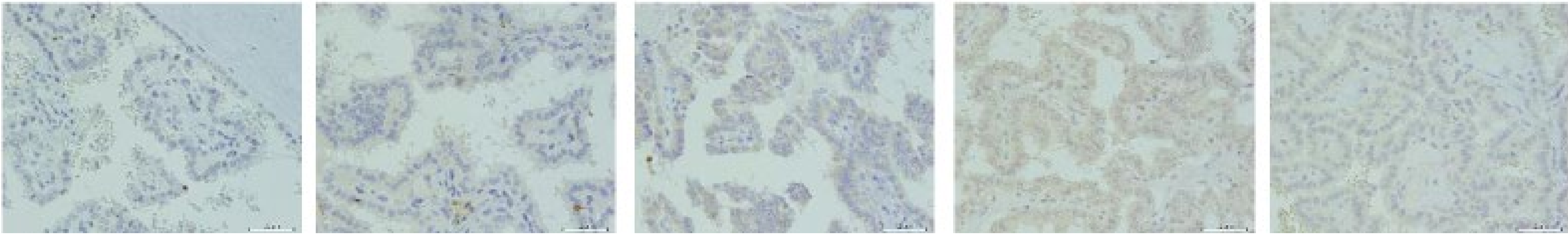

Figure S-2-20: HE and IHC images of PTC, sample 20

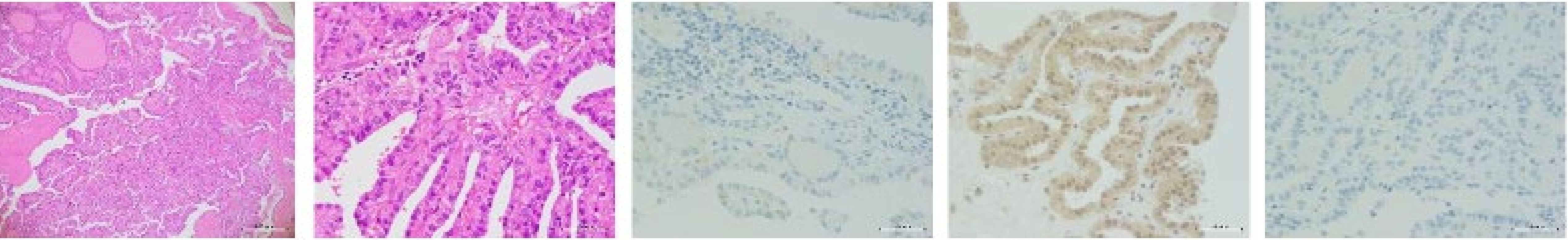

HE, low magnification

HE, high magnification

TLR2 score: 1x60+2x20 +3x40=200

TLR3 score: 3x100=300

TLR4 score: 0

TLR5 score: 0

TLR7 score: 1x50=50

TLR9 score: 1x20+2x20 +3x60=230

MyD88 score: 1x40+2x60=160

TRIF score: 1x50+2x50=150

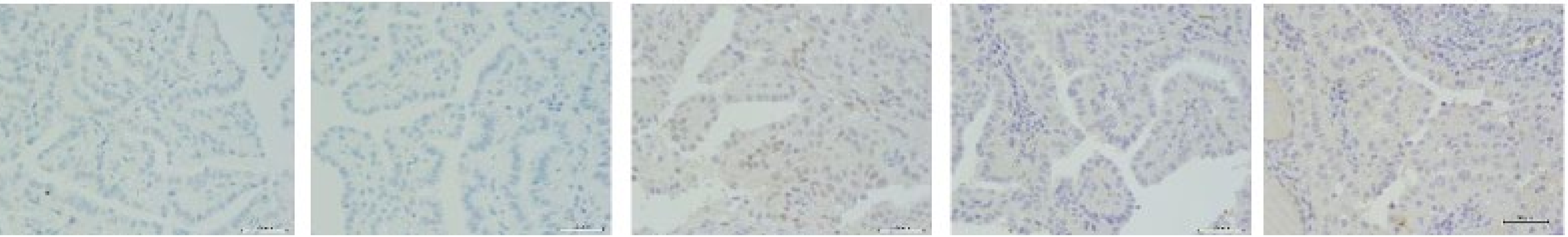

Figure S-2-21: HE and IHC images of PTC, sample 21

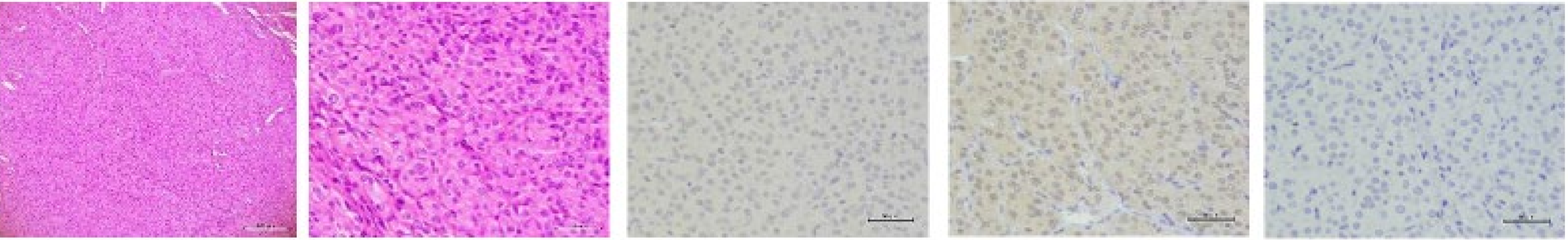

HE, low magnification

HE, high magnification

TLR2 score: 3x100=300

TLR3 score: 3x100=300

TLR4 score: 0

TLR5 score: 0

TLR7 score: 1x100=100

TLR9 score: 0

MyD88 score: 1x40+2x60=160

TRIF score: 1x20+2x40+3x40 =220

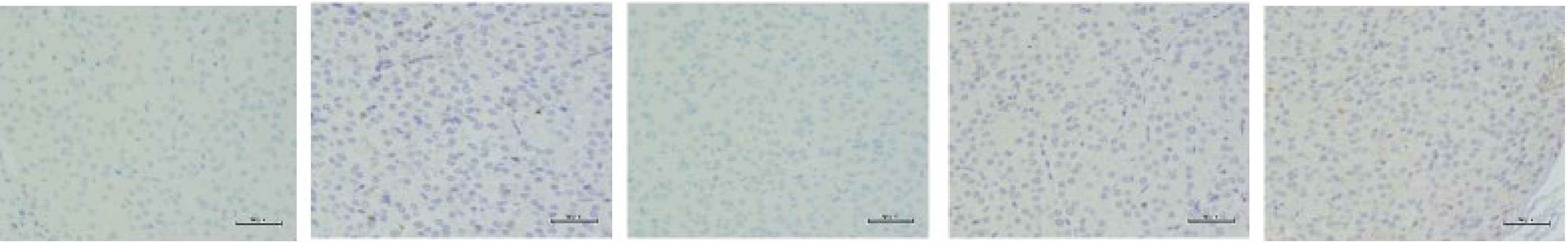

Figure S-2-22: HE and IHC images of PTC, sample 22

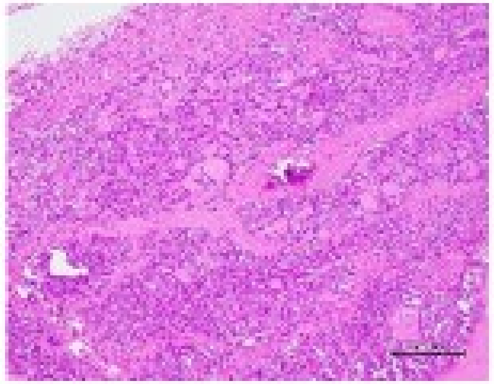
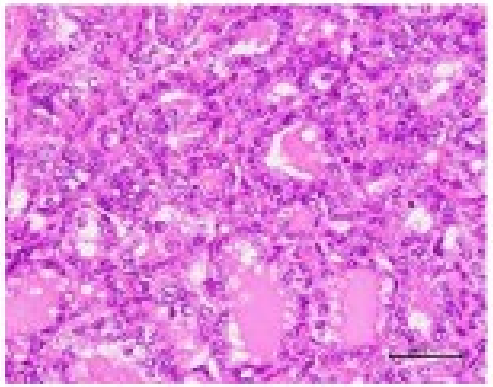
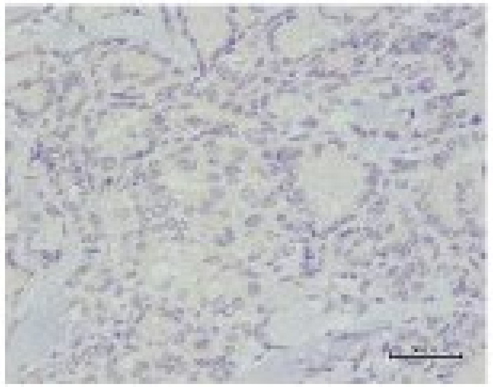
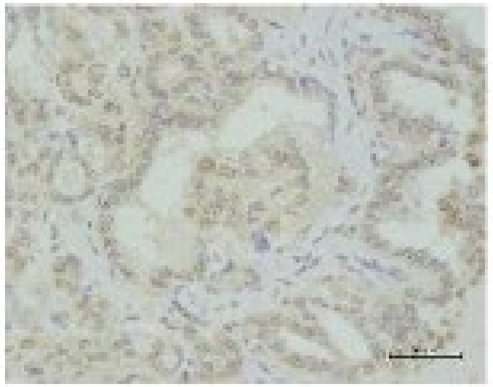
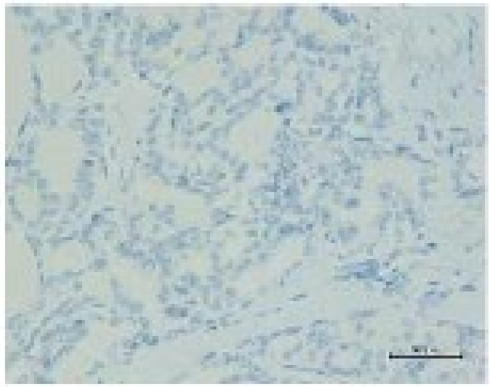

HE, low magnification

HE, high magnification

TLR2 score: 2x100=200

TLR3 score: 2x10+3x85=275

TLR4 score: 0

TLR5 score: 1x15+3x15=60

TLR7 score: 0

TLR9 score: 0

MyD88 score: 1x80=80

TRIF score: 2x100=200

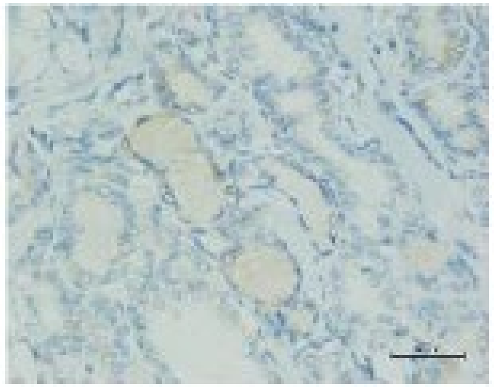
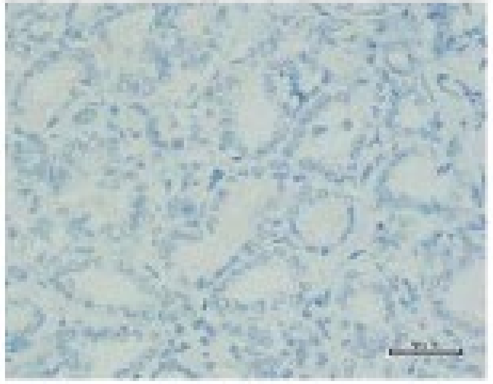
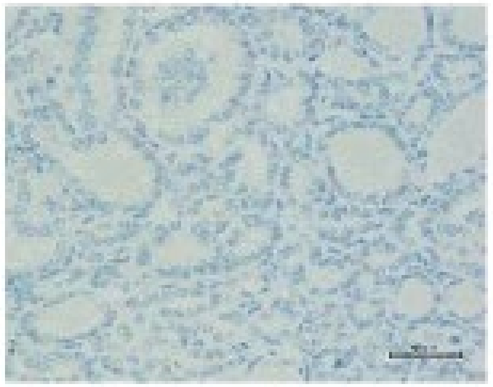
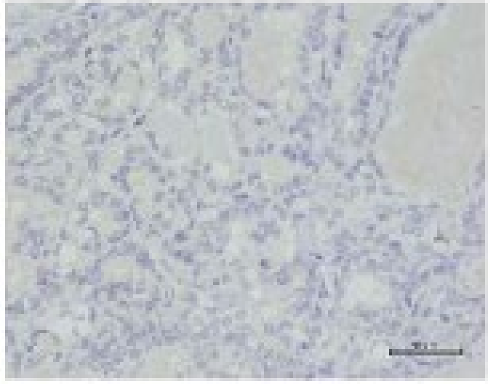
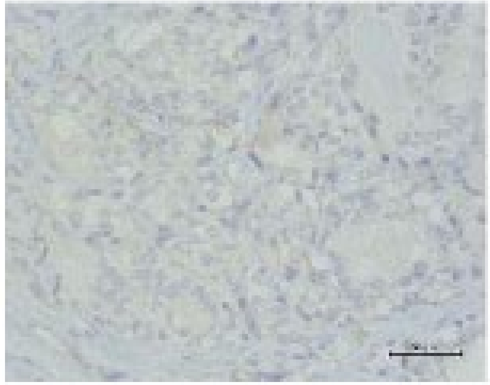

Figure S-2-23: HE and IHC images of PTC, sample 23

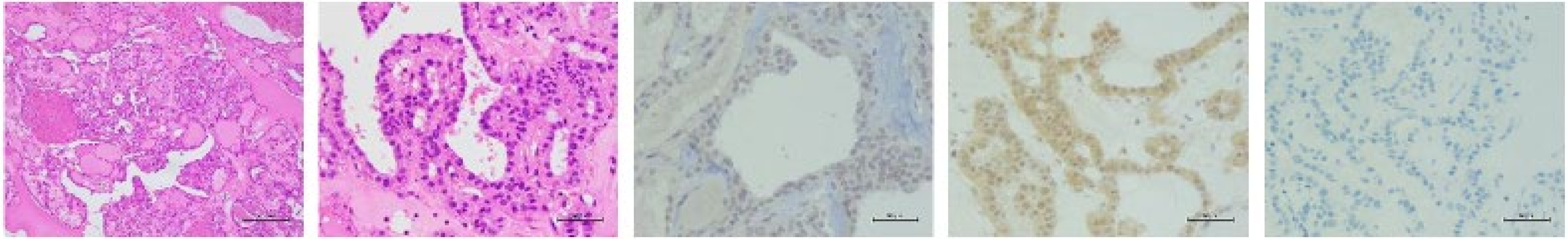

HE, low magnification

HE, high magnification

TLR2 score: 2x100=200

TLR3 score: 3x100=300

TLR4 score: 0

TLR5 score: 0

TLR7 score: 0

TLR9 score: 2x10+3x20=80

MyD88 score: 1x15+2x70 +3x15=200

TRIF score: 2x65+3x35=235

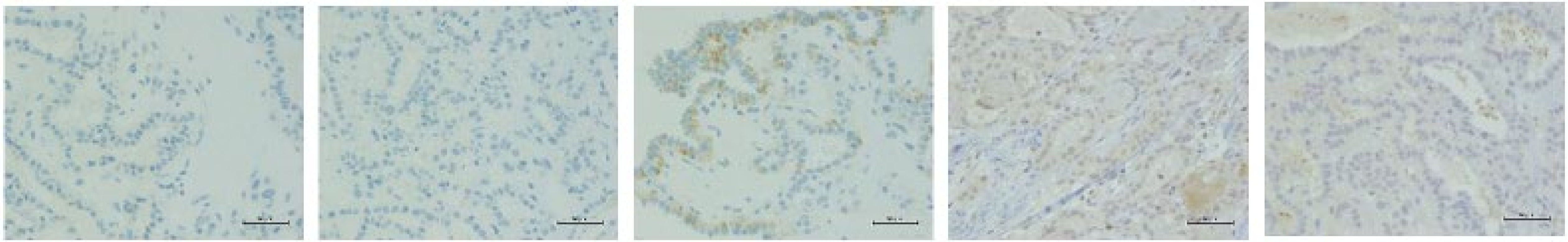

Figure S-2-24: HE and IHC images of PTC, sample 24

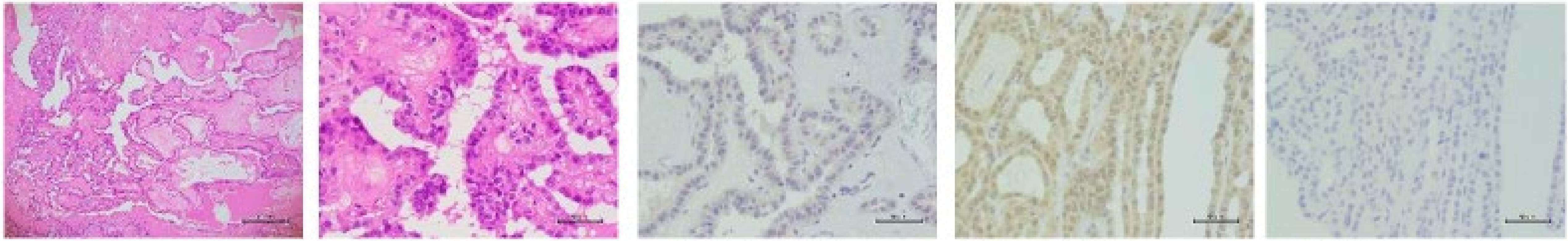

HE, low magnification

HE, high magnification

TLR2 score: 2x40+3x60=260

TLR3 score: 3x100=300

TLR4 score: 0

TLR5 score: 1x40+2x10=60

TLR7 score: 0

TLR9 score: 1x50+2x20+3x20=150

MyD88 score: 1x50+2x50=150

TRIF score: 1x50+2x50=150

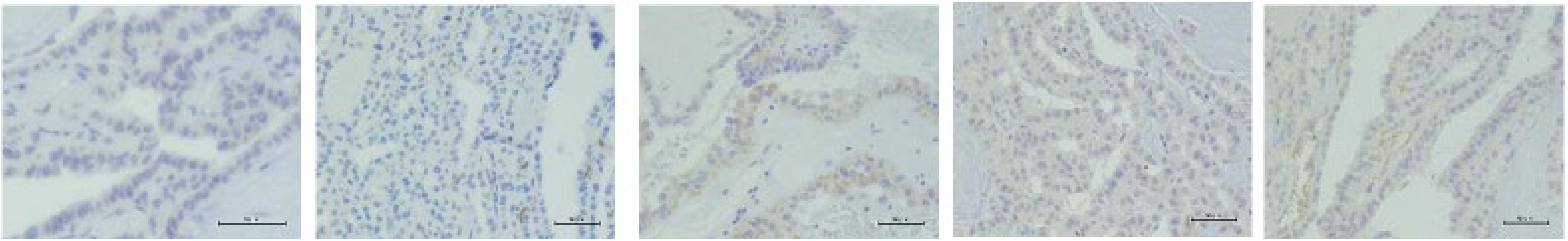

Figure S-2-25: HE and IHC images of PTC, sample 25

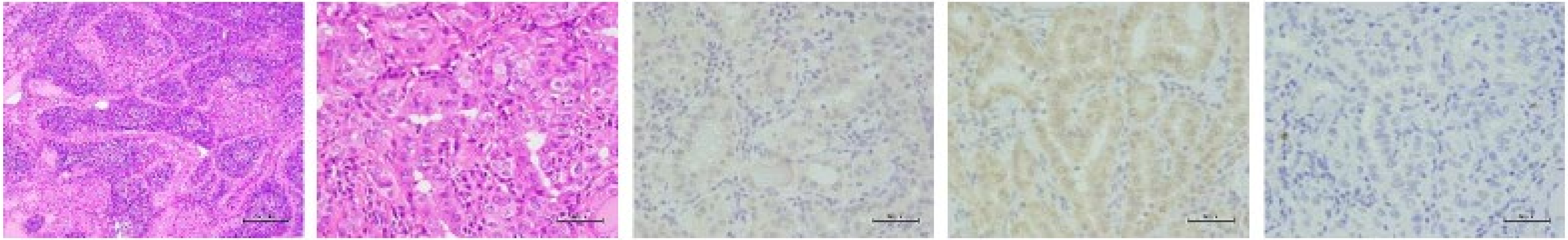

HE, low magnification

HE, high magnification

TLR2 score: 2x50+3x50 =250

TLR3 score: 3x100=300

TLR4 score: 0

TLR5 score: 0

TLR7 score: 1x80=80

TLR9 score: 1x20+2x50 +3x5=135

MyD88 score: 3x100=300

TRIF score: 1x50+2x50=150

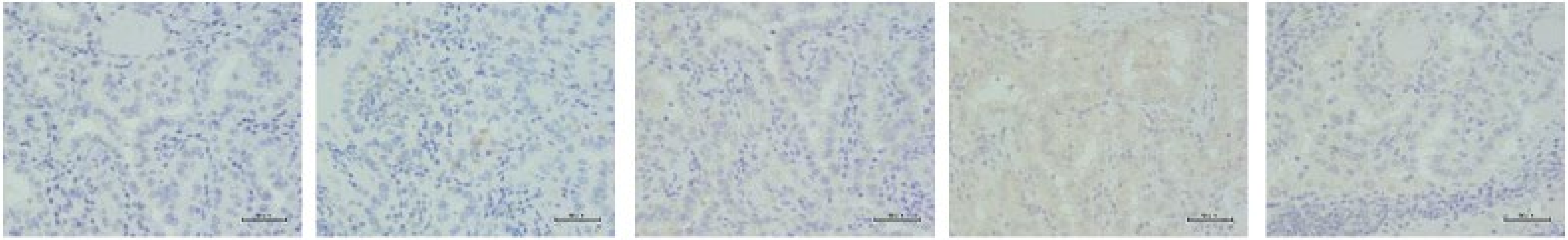

Figure S-3-1: HE and IHC images of ATC, sample 1

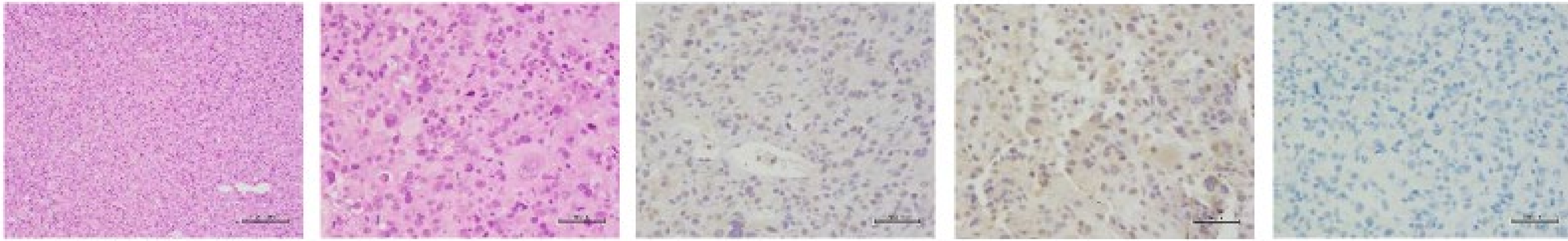

HE, low magnification

HE, high magnification

TLR2 score: 1x30+2x20=70

TLR3 score: 1x20+2x40 +3x25=175

TLR4 score: 0

TLR5 score: 1x40+2x60=160

TLR7 score: 1x60+2x30=120

TLR9 score: 1x70+2x10 +3x5=105

MyD88 score: 1x60+2x40=140

TRIF score: 1x20+2x40 +3x40=220

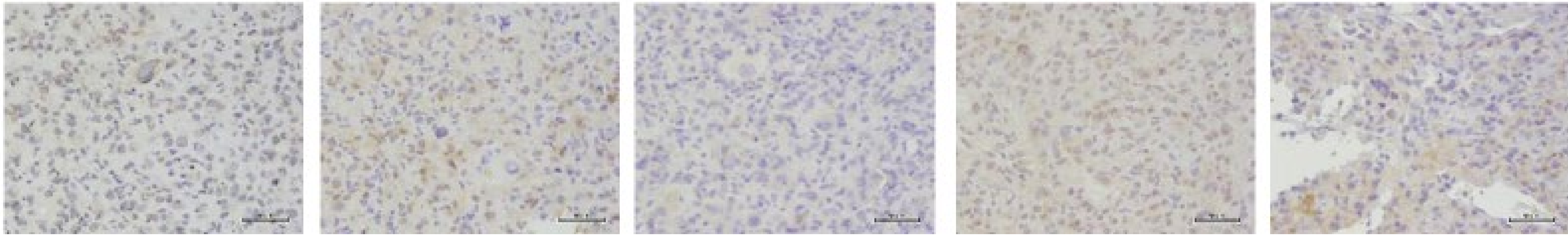

Figure S-3-2: HE and IHC images of ATC, sample 2

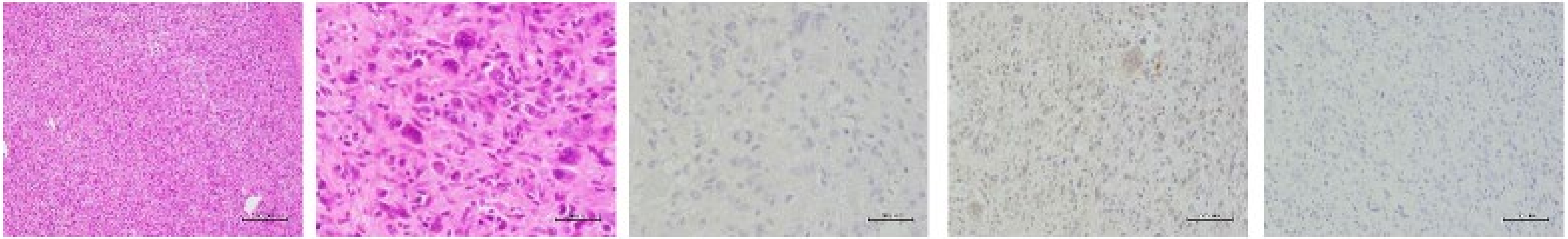

HE, low magnification

HE, high magnification

TLR2 score: 1x70=70

TLR3 score: 1x50+2x30+3x10 0=140

TLR4 score: 0

TLR5 score: 1x2=2

TLR7 score: 1x15=15

TLR9 score: 1x10=10

MyD88 score: 1x40=40

TRIF score: 2x50=100

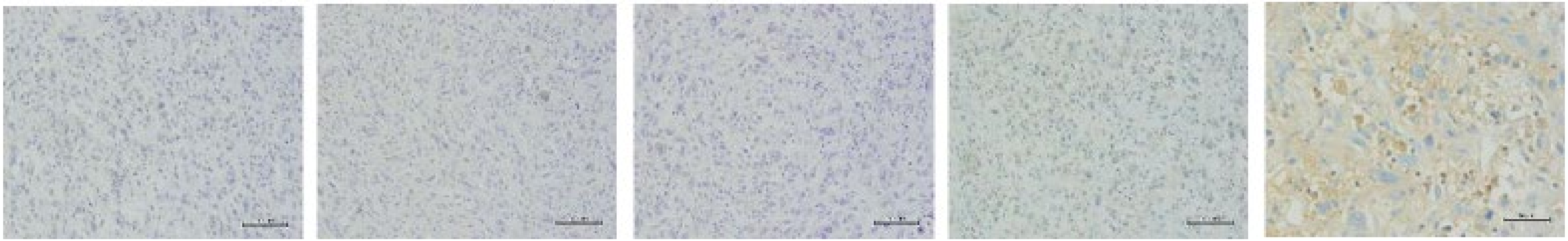

Figure S-3-3: HE and IHC images of ATC, sample 3

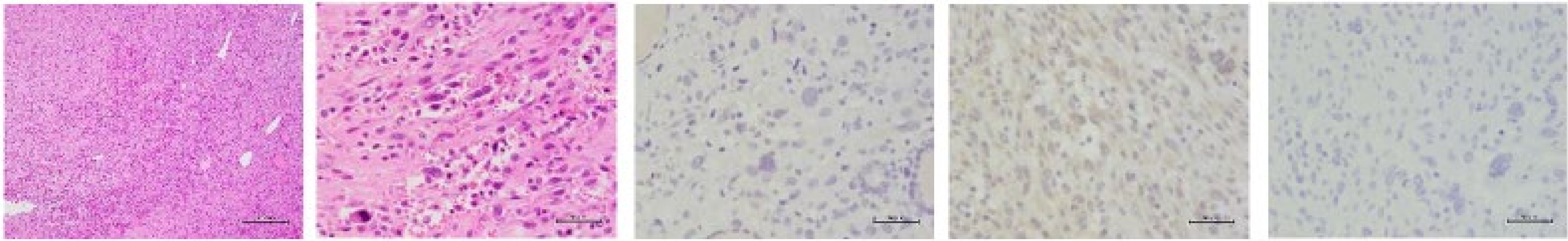

HE, low magnification

HE, high magnification

TLR2 score: 1x30+2x20=70

TLR3 score: 1x20+2x40 +3x40=220

TLR4 score: 0

TLR5 score: 1x5=5

TLR7 score: 1x20=20

TLR9 score: 1x2=2

MyD88 score: 1x50=50

TRIF score: 1x10=10

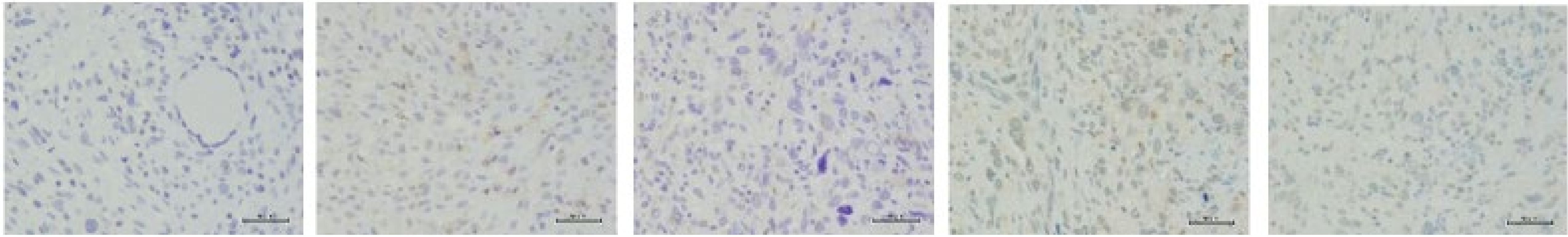

Figure S-3-4: HE and IHC images of ATC, sample 4

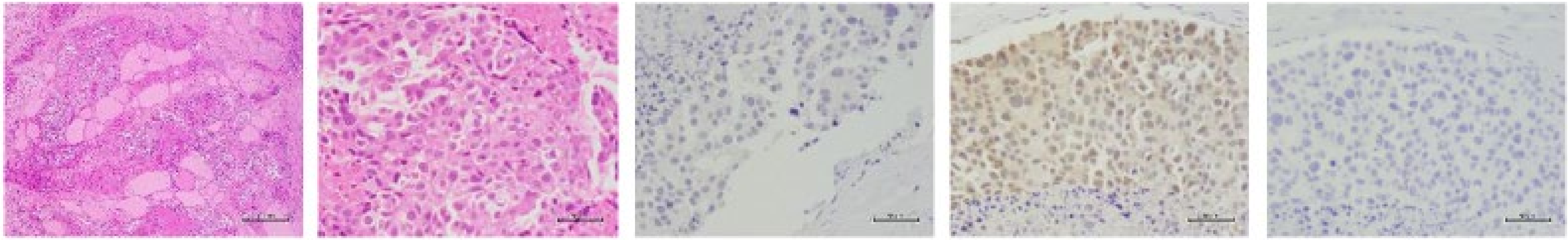

HE, low magnification

HE, high magnification

TLR2 score: 1x20=20

TLR3 score: 1x10+2x20+3x40 =270

TLR4 score: 0

TLR5 score: 1x5=5

TLR7 score: 1x25=25

TLR9 score: 0

MyD88 score: 1x35=35

TRIF score: 1x20+2x20=60

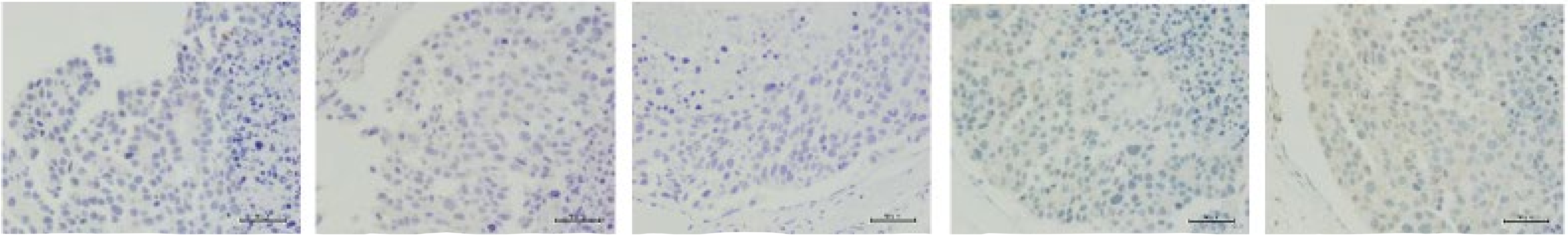

Figure S-3-5: HE and IHC images of ATC, sample 5

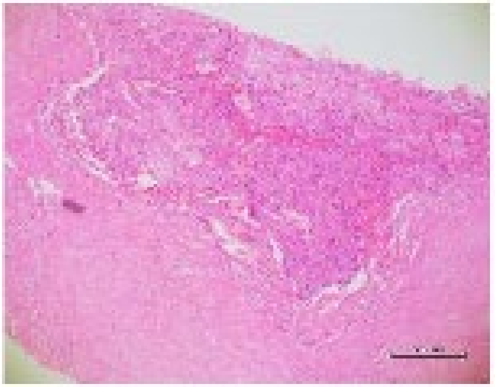

HE, low magnification

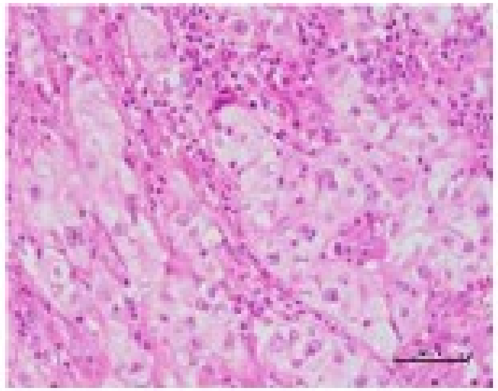

HE, high magnification

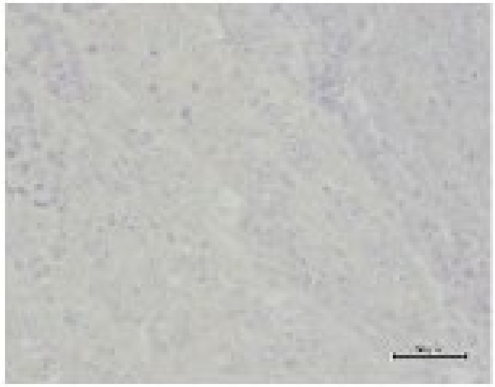

TLR2 score: 1x45+2x45=135

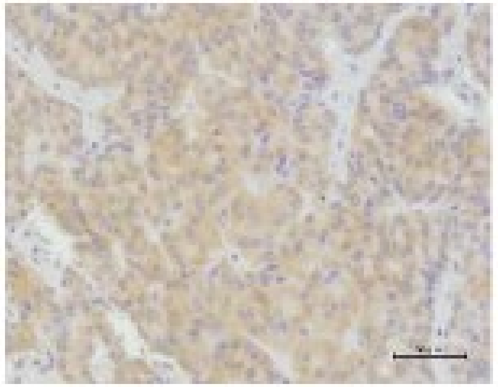

TLR3 score: 3x100=300

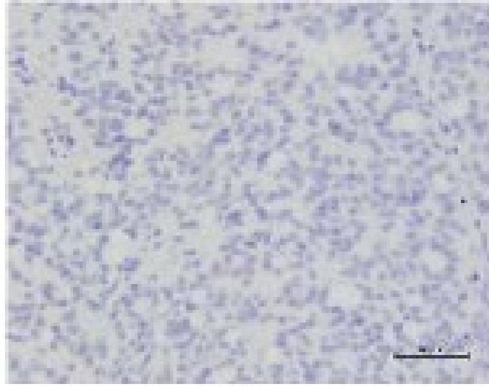

TLR4 score: 0

TLR5 score: 1x40=40

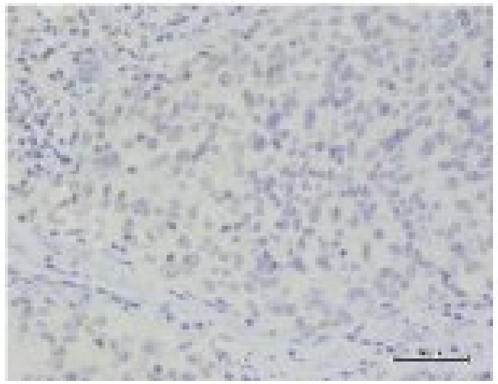

TLR7 score: 1x5=5

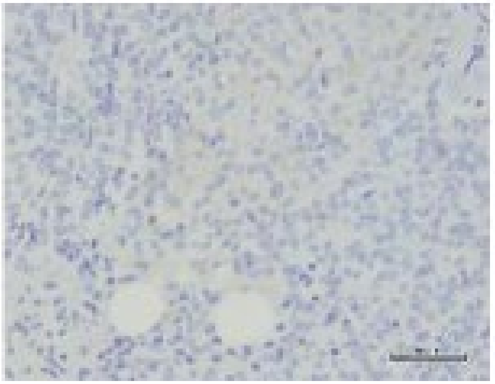

TLR9 score: 1x20+2x30 +3x30=170

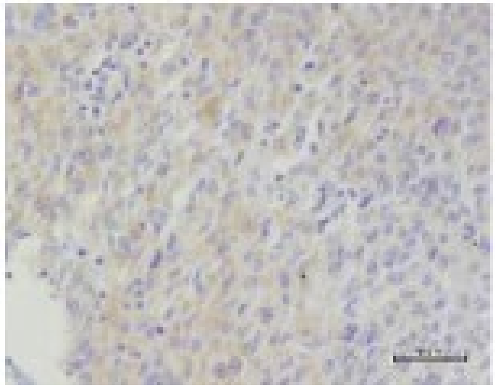

MyD88 score: 1x20+2x35 +3x5=105

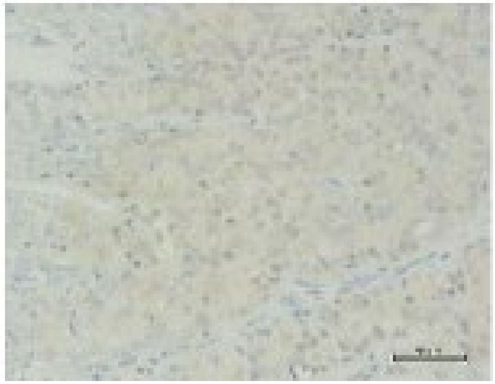

TRIF score: 1x10+3x40=130

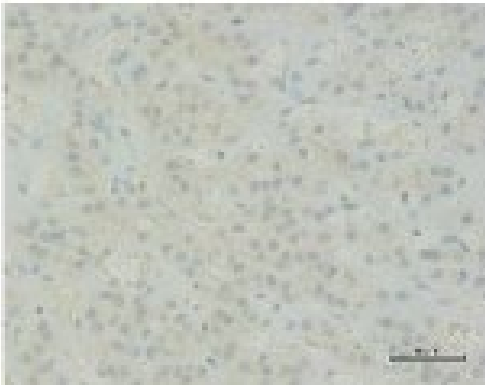

Figure S-3-6: HE and IHC images of ATC, sample 6

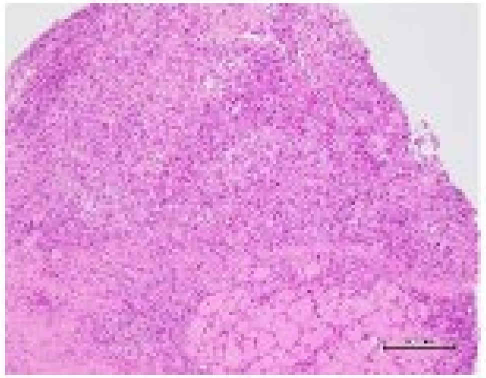

HE, low magnification

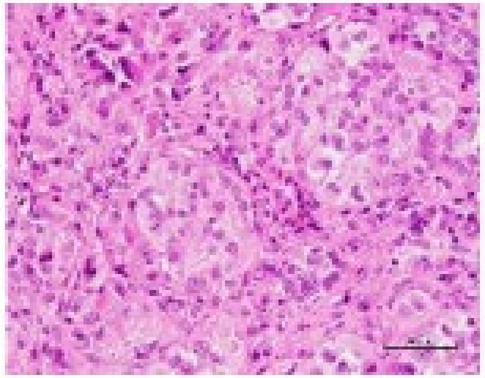

HE, high magnification

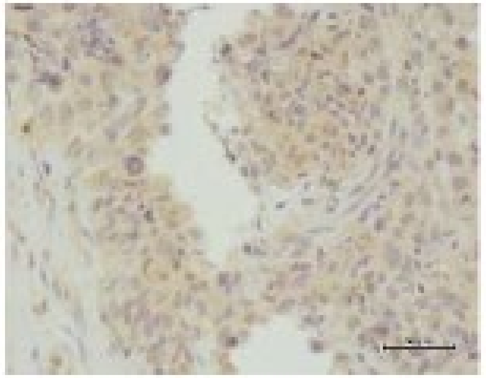

TLR2 score: 3x100=300

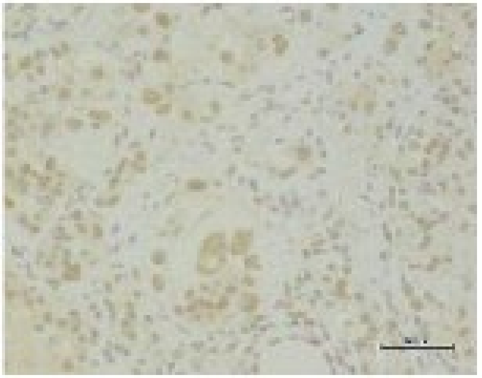

TLR3 score: 2x10+3x90=290

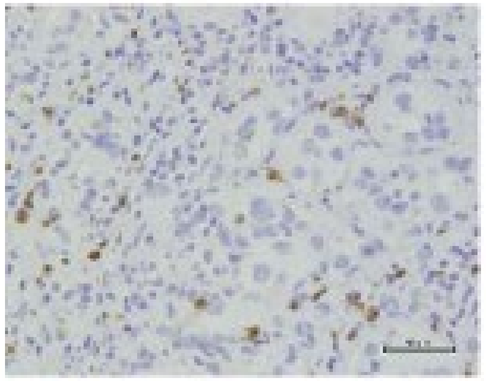

TLR4 score: 0

TLR5 score: 1x35=35

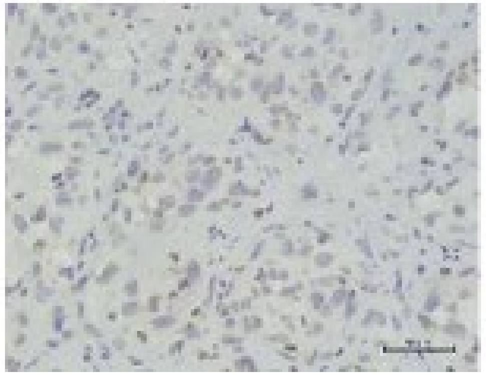

TLR7 score: 0

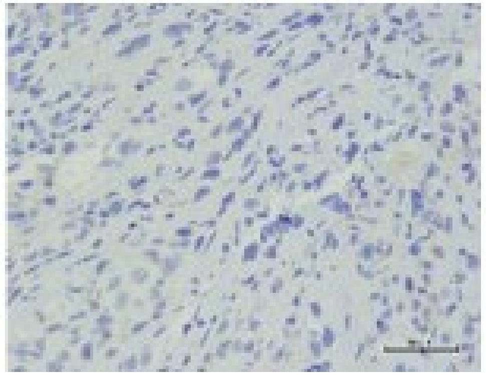

TLR9 score: 1x35+2x35=105

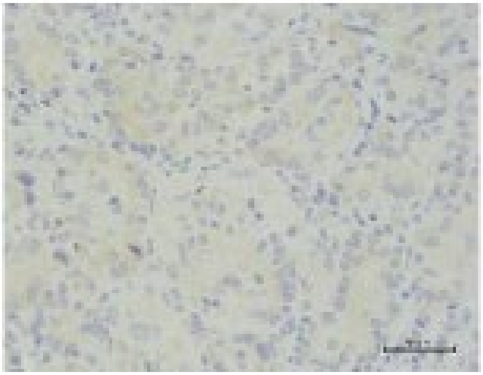

MyD88 score: 2x30+3x45=195

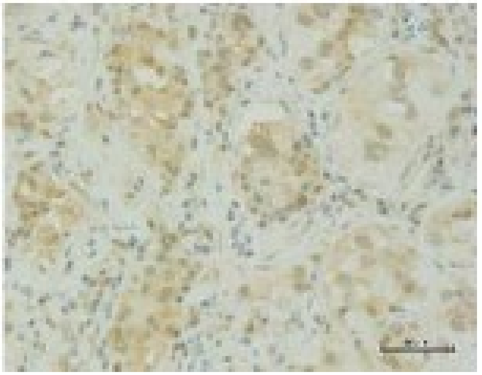

TRIF score: 1x75+2x20=115

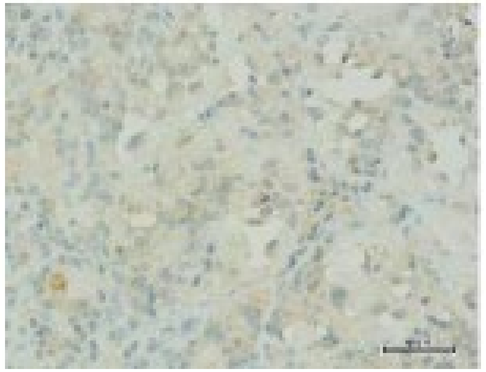

Figure S-3-7: HE and IHC images of ATC, sample 7

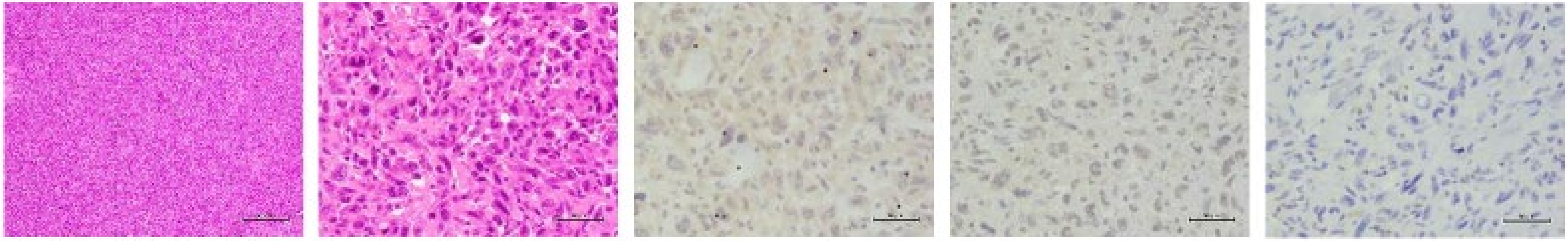

HE, low magnification

HE, high magnification

TLR2 score: 2x50+3x50 =250

TLR3 score: 1x40+2x60=160

TLR4 score: 0

TLR5 score: 1x15=15

TLR7 score: 1x5=5

TLR9 score: 1x10=10

MyD88 score: 1x85=85

TRIF score: 1x85=85

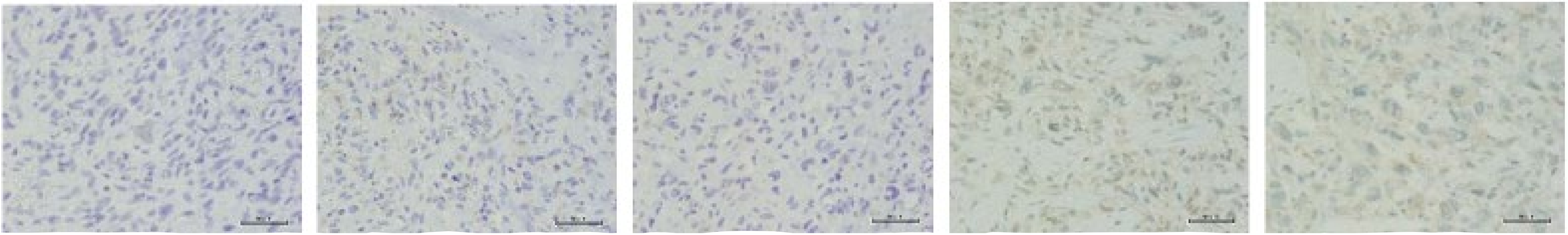

Figure S-3-8: HE and IHC images of ATC, sample 8

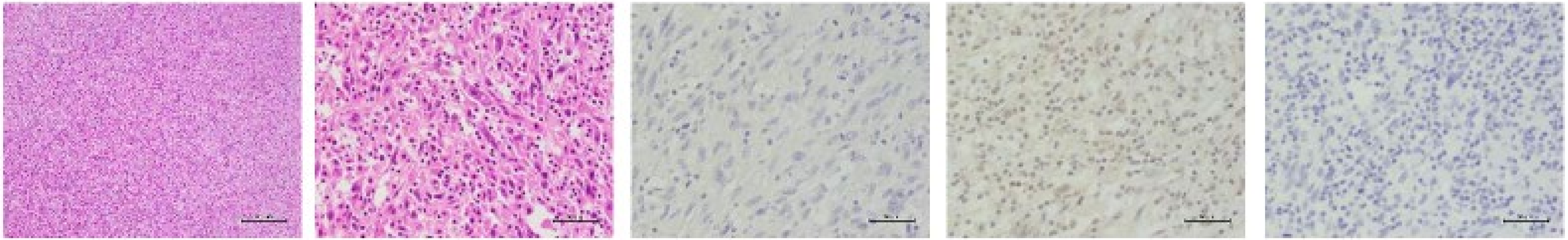

HE, low magnification

HE, high magnification

TLR2 score: 1x30=30

TLR3 score: 1x35+2x60=155

TLR4 score: 0

TLR5 score: 1x25=25

TLR7 score: 1x10=10

TLR9 score: 1x65=65

MyD88 score: 2x50+3x40=220

TRIF score: 1x70=70

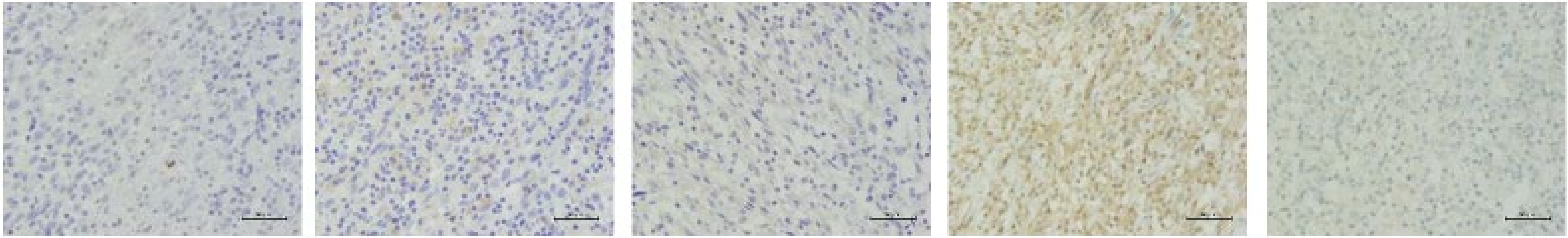

Figure S-3-9: HE and IHC images of ATC, sample 9

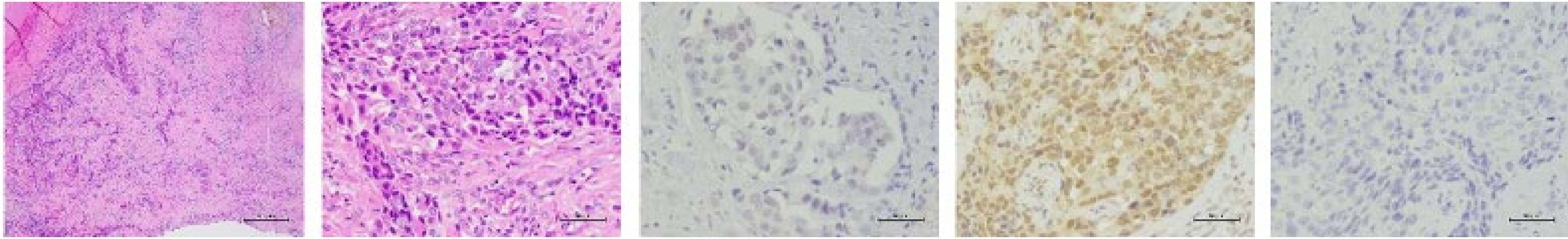

HE, low magnification

HE, high magnification

TLR2 score: 1x30=30

TLR3 score: 3x100=300

TLR4 score: 0

TLR5 score: 1x75+2x5=85

TLR7 score: 0

TLR9 score: 3x100=300

MyD88 score: 2x45+3x45=225

TRIF score: 1x50+2x50=150

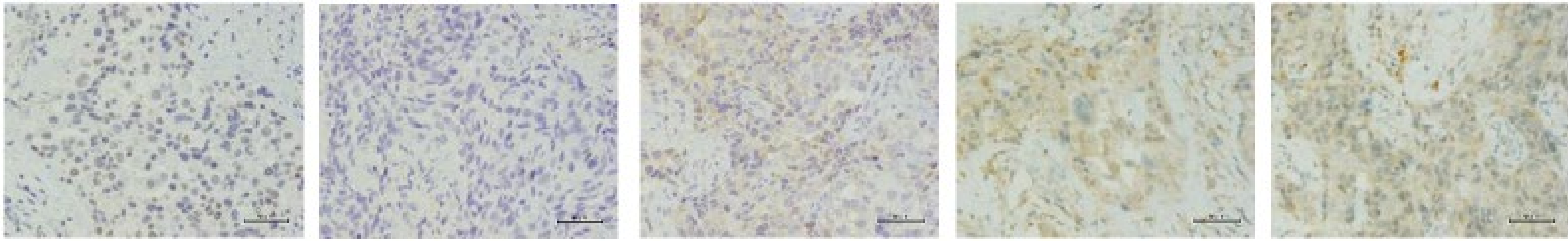

Figure S-3-10: HE and IHC images of ATC, sample 10

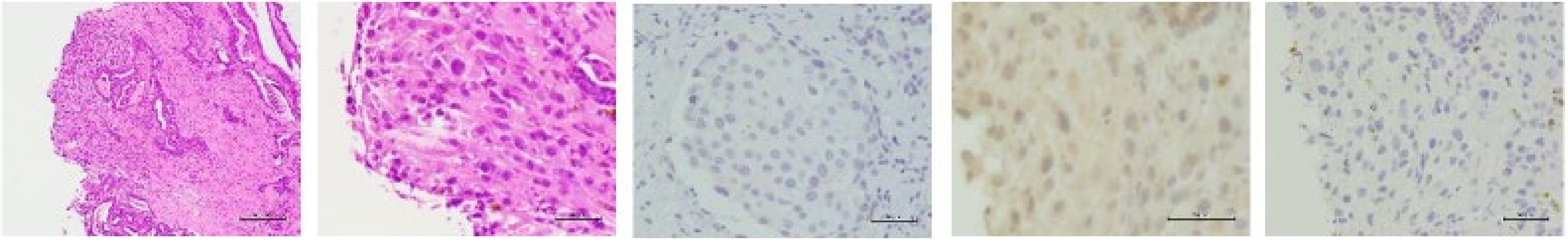

HE, low magnification

HE, high magnification

TLR2 score: 1x80=80

TLR3 score: 2x100=200

TLR4 score: 0

TLR5 score: 0

TLR7 score: 0

TLR9 score: 1x50=50

MyD88 score: 3x100=300

TRIF score: 1x10+2x10 +3x70=240

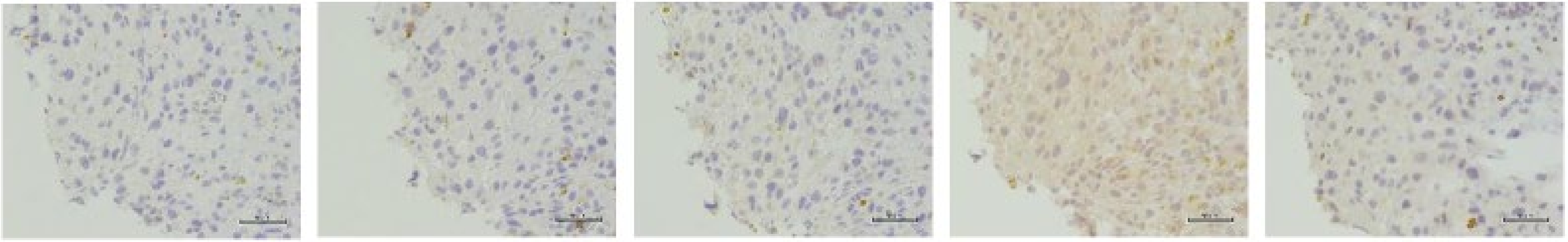

Figure S-3-11: HE and IHC images of ATC, sample 11

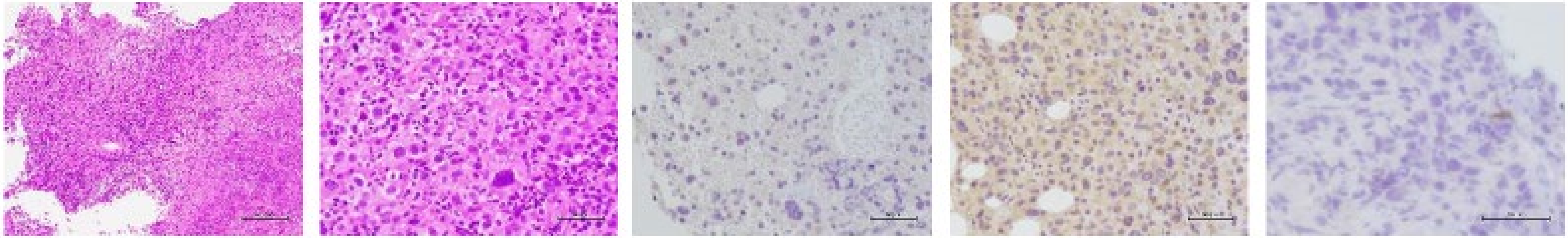

HE, low magnification

HE, high magnification

TLR2 score: 2x80=160

TLR3 score: 3x100=300

TLR4 score: 0

TLR5 score: 1x50=50

TLR7 score: 1x20=20

TLR9 score: 1x30+2x50=130

MyD88 score: 2x30+3x70=270

TRIF score: 3x90=270

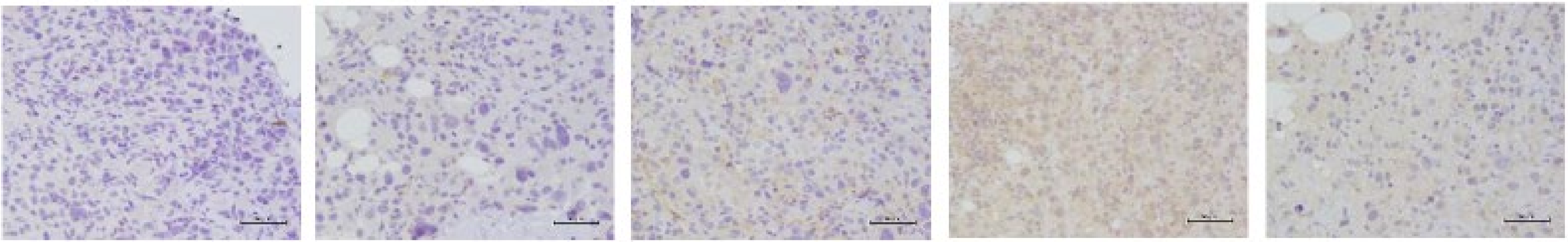

Supplement: Supplementary Materials — Figure S-1-1: HE and IHC images of NH, sample 1. Figure S-1-2: HE and IHC images of NH, sample 2. Figure S-1-3: HE and IHC images of NH, sample 3. Figure S-1-4: HE and IHC images of NH, sample 4. Figure S-1-5: HE and IHC images of NH, sample 5. Figure S-1-6: HE and IHC images of NH, sample 6. Figure S-1-7: HE and IHC images of NH, sample 7. Figure S-1-8: HE and IHC images of NH, sample 8. Figure S-2-1: HE and IHC images of PTC, sample 1. Figure S-2-2: HE and IHC images of PTC, sample 2. Figure S-2-3: HE and IHC images of PTC, sample 3. Figure S-2-4: HE and IHC images of PTC, sample 4. Figure S-2-5: HE and IHC images of PTC, sample 5. Figure S-2-6: HE and IHC images of PTC, sample 6. Figure S-2-7: HE and IHC images of PTC, sample 7. Figure S-2-8: HE and IHC images of PTC, sample 8. Figure S-2-9: HE and IHC images of PTC, sample 9. Figure S-2-10: HE and IHC images of PTC, sample 10. Figure S-2-11: HE and IHC images of PTC, sample 11. Figure S-2-12: HE and IHC images of PTC, sample 12. Figure S-2-13: HE and IHC images of PTC, sample 13. Figure S-2-14: HE and IHC images of PTC, sample 14. Figure S-2-15: HE and IHC images of PTC, sample 15. Figure S-2-16: HE and IHC images of PTC, sample 16. Figure S-2-17: HE and IHC images of PTC, sample 17. Figure S-2-18: HE and IHC images of PTC, sample 18. Figure S-2-19: HE and IHC images of PTC, sample 19. Figure S-2-20: HE and IHC images of PTC, sample 20. Figure S-2-21: HE and IHC images of PTC, sample 21. Figure S-2-22: HE and IHC images of PTC, sample 22. Figure S-2-23: HE and IHC images of PTC, sample 23. Figure S-2-24: HE and IHC images of PTC, sample 24. Figure S-2-25: HE and IHC images of PTC, sample 25. Figure S-3-1: HE and IHC images of ATC, sample 1. Figure S-3-2: HE and IHC images of ATC, sample 2. Figure S-3-3: HE and IHC images of ATC, sample 3. Figure S-3-4: HE and IHC images of ATC, sample 4. Figure S-3-5: HE and IHC images of ATC, sample 5. Figure S-3-6: HE and IHC images of ATC, sample 6. Figure S-3-7: HE and IHC i [file 4226491.f1.pdf]
